# YKT6 Promotes Bladder Cancer Progression by Stabilizing β‐catenin Through USP7‐Mediated Deubiquitination

**DOI:** 10.1002/advs.202507166

**Published:** 2025-11-26

**Authors:** Sheng Tu, Wenzhi Du, Yongwen Luo, Jiageng Shi, Tianyun Liu, Meng Ji, Kangping Xiong, Siming Chen, Fenfang Zhou, Mingxing Li, Jingtian Yu, Gang Wang, Lingao Ju, Yi Zhang, Yu Xiao, Xinghuan Wang, Kaiyu Qian

**Affiliations:** ^1^ Department of Urology Zhongnan Hospital of Wuhan University Wuhan 430071 China; ^2^ Hubei Key Laboratory of Urological Diseases Zhongnan Hospital of Wuhan University Wuhan 430071 China; ^3^ Department of Urology The First Affiliated Hospital of Shandong First Medical University & Shandong Provincial Qianfoshan Hospital Jinan Shandong 250014 China; ^4^ Department of Laboratory Medicine Zhongnan Hospital of Wuhan University Wuhan 430071 China; ^5^ Department of Radiology Zhongnan Hospital of Wuhan University Wuhan 430071 China; ^6^ Department of Biological Repositories Human Genetic Resources Preservation Center of Hubei Province Zhongnan Hospital of Wuhan University Wuhan 430071 China; ^7^ Institute for Human Genetics and Molecular Medicine Chinese Institutes for Medical Research Beijing 100069 China; ^8^ Wuhan Research Center for Infectious Diseases and Cancer Chinese Academy of Medical Sciences Wuhan 430071 China; ^9^ Medical Research Institute Frontier Science Center for Immunology and Metabolism Wuhan University Wuhan 430071 China

**Keywords:** bladder cancer, ubiquitination, USP7, Wnt/β‐catenin signaling, YKT6

## Abstract

Bladder cancer (BLCA) remains a highly lethal genitourinary malignancy with complex tumor biology and limited therapeutic strategies. This study investigates the oncogenic role of YKT6, a SNARE protein, in BLCA progression and molecular mechanisms. It is demonstrated that YKT6 is significantly upregulated in BLCA tissues and cell lines, correlating with advanced tumor grade, aggressive histology, and poor patient prognosis from public datasets and tissue microarray. Transcriptomic and functional analyses reveal that YKT6 promotes BLCA cell proliferation, migration, and metastasis both in vitro and in vivo. Mechanistically, YKT6 activates the Wnt/β‐catenin signaling pathway through a novel mechanism involving USP7‐mediated deubiquitination of β‐catenin. By recruiting USP7, YKT6 inhibits β‐catenin's proteasomal degradation, thereby stabilizing the protein and driving nuclear accumulation. This stabilization leads to increased expression of oncogenic target genes and induces epithelial‐mesenchymal transition (EMT). Pharmacological interventions demonstrate that Wnt signaling inhibition reverses YKT6‐driven malignant effects, while pathway activation restores tumor progression in YKT6‐silenced cells. USP7 knockdown abrogates YKT6‐mediated β‐catenin stabilization, confirming their functional interdependence. Clinically, the YKT6/USP7/β‐catenin axis strongly correlates with poor prognosis, providing a proof‐of‐concept for the druggability of this axis. The findings unveil YKT6 as a novel regulator of Wnt signaling through USP7‐dependent deubiquitination, offering insights for precision BLCA therapy.

## Graphical abstract

The SNARE protein YKT6 is upregulated in bladder cancer (BLCA), correlating with poor prognosis. YKT6 promotes tumor proliferation and metastasis by activating Wnt/β‐catenin signaling. It recruits Ubiquitin‐Specific Peptidase 7 (USP7) to deubiquitinate and stabilize β‐catenin, enhancing its nuclear accumulation and driving oncogenic gene expression. The YKT6/USP7/β‐catenin axis represents a potential therapeutic target in BLCA.

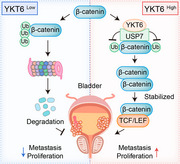

## 1 Introduction

Bladder cancer (BLCA) is a universal and highly malignant tumor, which ranks among the most prevalent malignancies in genitourinary tumors. Recent epidemiological data show that BLCA ranks as the 9th most prevalent cancer globally, with ≈613 thousand new cases and 220 thousand deaths.^[^ ^1^ ^]^ ≈75% of newly diagnosed BLCA are categorized as non‐muscle‐invasive bladder cancer (NMIBC), and the remainder are grouped into muscle‐invasive bladder cancer (MIBC).^[^ ^2^ ^]^ ≈69% of NMIBCs are characterized by low‐grade features,^[^ ^3^ ^]^ while the majority of muscle‐invasive or metastatic tumors exhibit high‐grade features.^[^ ^4^, ^5^ ^]^ The 5‐year recurrence rates of NMIBC vary from 31%–78%,^[^ ^6^ ^]^ and their progression rates span from 0.93% in low‐risk to 40% in very high‐risk groups.^[^ ^3^ ^]^ For MIBC patients receiving treatment, the 5‐year overall survival rate is ≈48%,^[^ ^4^ ^]^ but for metastatic patients, the rate drops to ≈5.5%.^[^ ^5^ ^]^ It is essential to uncover the molecular mechanisms of BLCA malignancy progression and explore more effective treatment strategies.

Soluble N‐ethylmaleimide‐sensitive factor attachment protein receptor (SNARE) proteins, within a conserved SNARE motif, mediate intracellular membrane fusion events.^[^ ^7^ ^]^ YKT6, a conserved SNARE, serves as a key mediator in membrane fusion and is associated with vesicular transit.^[^ ^8^ ^]^ Moreover, this evolutionarily conserved protein maintains remarkable structural similarity across diverse species, ranging from yeast to mammals.^[^ ^9^, ^10^ ^]^ The functional dynamics of YKT6 involve its existence in both membrane‐associated form and soluble cytoplasmic pools, and YKT6 undergoes cycling between membranes and cytosol for sequential vesicle docking and fusion events.^[^ ^11^ ^]^ Research on non‐small cell lung cancer (NSCLC) reports that YKT6 promotes both migration and invasion of lung cancer cells. Furthermore, analyses of clinical samples have revealed that high YKT6 expression is significantly associated with poor overall survival in NSCLC patients.^[^ ^12^, ^13^, ^14^ ^]^ Research on breast cancer has indicated that YKT6 is correlated with malignant phenotypes and triggers therapeutic resistance.^[^ ^15^, ^16^ ^]^ In addition, YKT6 promotes oral squamous cell carcinoma cell lines' migration and invasion in vitro and correlates aberrant immune status marked by dysfunctional CD8^+^ T cells.^[^ ^17^ ^]^ In pancreatic cancer, YKT6 is involved in the transportation of multivesicular bodies regulated by the long non‐coding RNA PVT1.^[^ ^18^ ^]^ A pan‐cancer bioinformatics analysis of YKT6 reveals that YKT6 is elevated in BLCA and is predicted poor prognosis.^[^ ^19^ ^]^ Despite emerging interest in YKT6 oncogenic potential, its context‐dependent behavior in BLCA remains unclear, highlighting an urgent need for investigation to uncover its biological function and therapeutic potential.

As an evolutionary conservative pathway, the Wnt signal transduction cascade regulates a multitude of critical embryonic and somatic processes, such as organogenesis, cell fate determination, and various pathological conditions.^[^ ^20^ ^]^ It is also strongly implicated in cancer progression, including the cancer stem cell maintenance, metastasis initiation, and other mechanisms driving tumor progression.^[^ ^21^ ^]^ β‐catenin, the pathway's key downstream modulator, is dynamically regulated by phosphorylation, ubiquitination, and degradation in the cytoplasmic β‐catenin destruction complex. This complex, comprising Axin, APC, GSK3, and CK1, coordinates sequential phosphorylation events: CK1‐mediated priming phosphorylation at Ser45 precedes GSK3β‐dependent phosphorylation at Thr41/Ser37/Ser33 residues, ultimately triggering ubiquitination‐mediated proteasomal targeting of β‐catenin.^[^ ^22^, ^23^, ^24^ ^]^ Wnt ligand binding to LRP5/6 co‐receptor initiates intracellular signaling through LRP5/6 phosphorylation, thereby blocking the function of β‐catenin degradation machinery and stabilizing cytosolic β‐catenin. Subsequent nuclear translocation enables β‐catenin to form transcriptional complexes with T cell factor/lymphocyte enhancer factor‐1 (TCF/LEF), ultimately inducing the expression of β‐catenin target genes, such as *MYC*.^[^ ^25^, ^26^, ^27^, ^28^ ^]^

This study demonstrates that upregulated YKT6 predicts BLCA patients’ poor prognosis. Functionally, YKT6 promotes BLCA proliferation and metastasis via modulating the Wnt/β‐catenin signaling pathway activity in vitro and in vivo. Mechanistically, YKT6 interacts with USP7 to facilitate β‐catenin deubiquitination and stabilization, thereby sustaining its oncogenic activity and driving BLCA progression. These findings uncover the YKT6/USP7/β‐catenin regulatory axis in BLCA, offering promising insights for BLCA management.

## 2 Results

### 2.1 YKT6 Upregulation Predicts Poor Prognosis in BLCA

**Figure 1:**
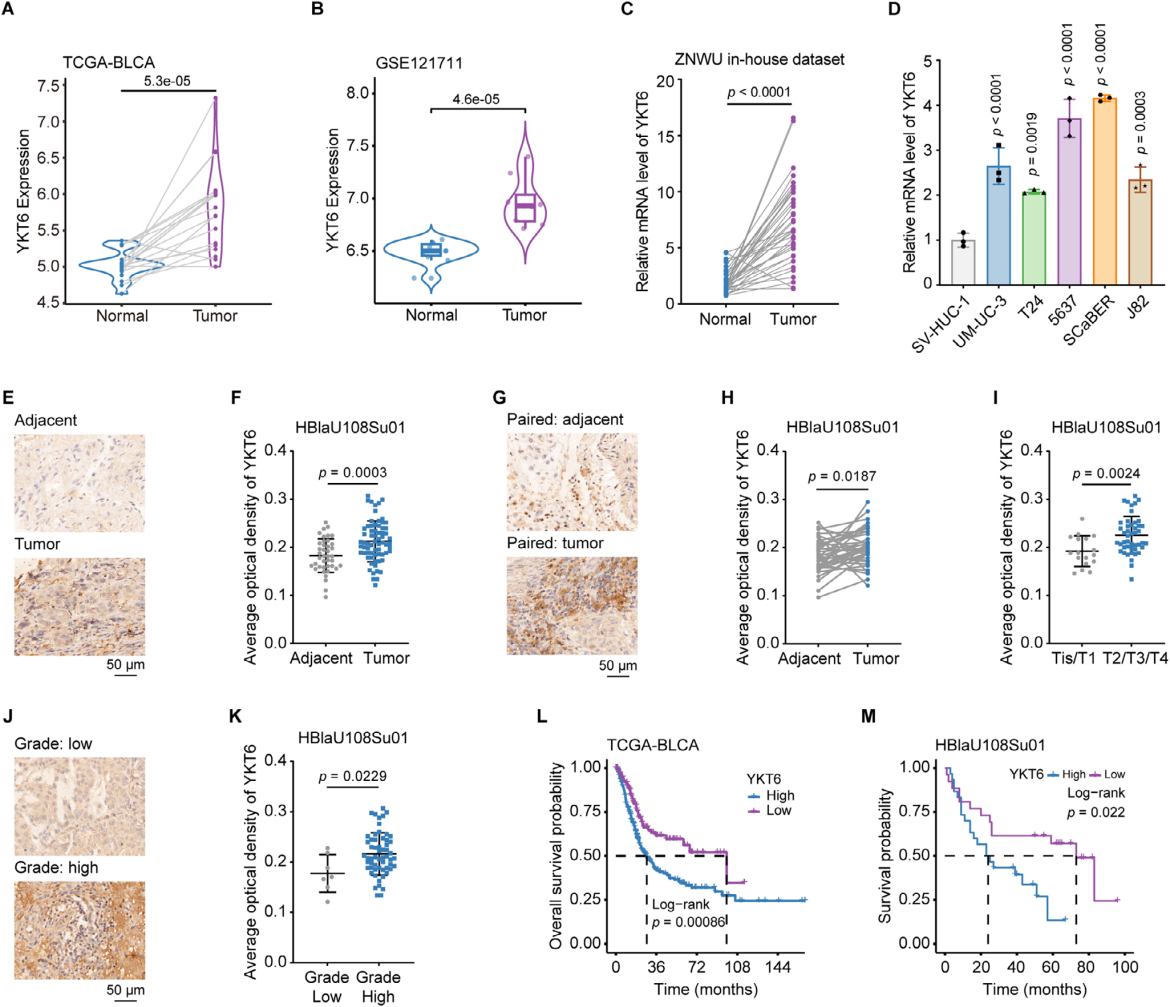
YKT6 upregulation predicts poor prognosis in BLCA. A) Relative YKT6 expression in adjacent and BLCA tissues from the TCGA database (n = 19). B) Relative YKT6 expression level in GSE121711 cohort (Normal: n = 10; Tumor: n = 8). C) qRT‐PCR validation of YKT6 upregulation in BLCA tissues (n = 36) from ZNWU in‐house dataset. D) Elevated YKT6 mRNA levels in BLCA cell lines compared to normal urothelial cells (SV‐HUC‐1, n = 3 per group). E,F) Representative IHC images (E) and statistical chart (F) of YKT6 proteins in adjacent (n = 40) and tumor (n = 68) tissue from HBlaU108Su01 cohort. G,H) Representative IHC images (G) and statistical chart (H) of YKT6 in paired adjacent (n = 40) and tumor (n = 40) tissue of BLCA from HBlaU108Su01 cohort. I) Statistical chart of YKT6 protein expression in different T stages (Tis/T1: n = 17; T2/T3/T4: n = 46) from HBlaU108Su01 cohort. J,K) Representative IHC images (J) and statistical chart (K) of YKT6 protein in low (n = 7) and high (n = 58) grade of BLCA from HBlaU108Su01 cohort. L,M) Kaplan‐Meier survival curves showing reduced overall survival in BLCA patients with high YKT6 expression in the TCGA database (L, n = 402) and HBlaU108Su01 cohort (M, n = 56). Data are presented as mean values ± SD; Wilcoxon test (A,B); two‐tailed paired Student's t‐test (C,H); two‐tailed unpaired Student's t‐test (F,I,K); one‐way ANOVA with Dunnett's multiple comparisons test (D); log‐rank test for survival analysis (L,M).

To evaluate the clinical significance of YKT6 in BLCA, we examined transcriptomic data from TCGA‐BLCA, GSE13507, GSE121711, and GSE31684 cohorts. YKT6 mRNA expression was elevated in BLCA relative to normal counterparts (**Figure** **1**A,B; Figure S1A, Supporting Information). In addition, qRT‐PCR validation in 36 paired BLCA and adjacent normal tissues from Zhongnan Hospital of Wuhan University (ZNWU) in‐house dataset confirmed this differential expression pattern of YKT6 (Figure 1C; Table S1, Supporting Information). Consistently, BLCA cell lines exhibited higher YKT6 mRNA levels than normal urothelial cells (Figure 1D). In addition, IHC staining of YKT6 from BLCA tissue microarray indicated that the YKT6 protein level was higher in BLCA tissues than in adjacent tissues (Figure 1E–H). Further investigation of YKT6 in BLCA indicated that YKT6 mRNA expression correlated with higher T stage (Ta/T1 vs T2/T3/T4, Figure S1B, Supporting Information), advanced tumor grade (high vs low grade, Figure S1C, Supporting Information), aggressive histology (non‐papillary vs papillary BLCA, Figure S1D, Supporting Information), and higher clinical stage (stage III‐IV vs stage I‐II, Figure S1E, Supporting Information). BLCA tissue microarray data confirmed that the YKT6 protein level was elevated in tumors with higher T stage and advanced grade (Figure 1I–K). The above data indicated a positive relationship between YKT6 expression level and malignancy of BLCA. Receiver operating characteristic (ROC) analysis revealed robust diagnostic potential for YKT6, with areas under the curve (AUC) of 0.824 (TCGA‐BLCA, Figure S1F, Supporting Information) and 0.810 (GSE13507, Figure S1G, Supporting Information), suggesting its utility as a diagnostic biomarker. Univariate Cox regression identified YKT6 as a prognostic risk factor for overall survival (OS), disease‐specific survival (DSS), and progression‐free interval (PFI) (Figure S1H, Supporting Information). Finally, survival analysis based on TCGA‐BLCA, GSE13507, and HBlaU108Su01 cohorts further confirmed that patients with elevated YKT6 expression had bad prognoses (Figure 1L,M; Figure S1I–L, Supporting Information). Therefore, these findings establish YKT6 as a biomarker linked to BLCA aggressiveness and unfavorable clinical outcomes.

### 2.2 YKT6 Knockdown Inhibits BLCA Proliferation In Vitro and In Vivo

**Figure 2:**
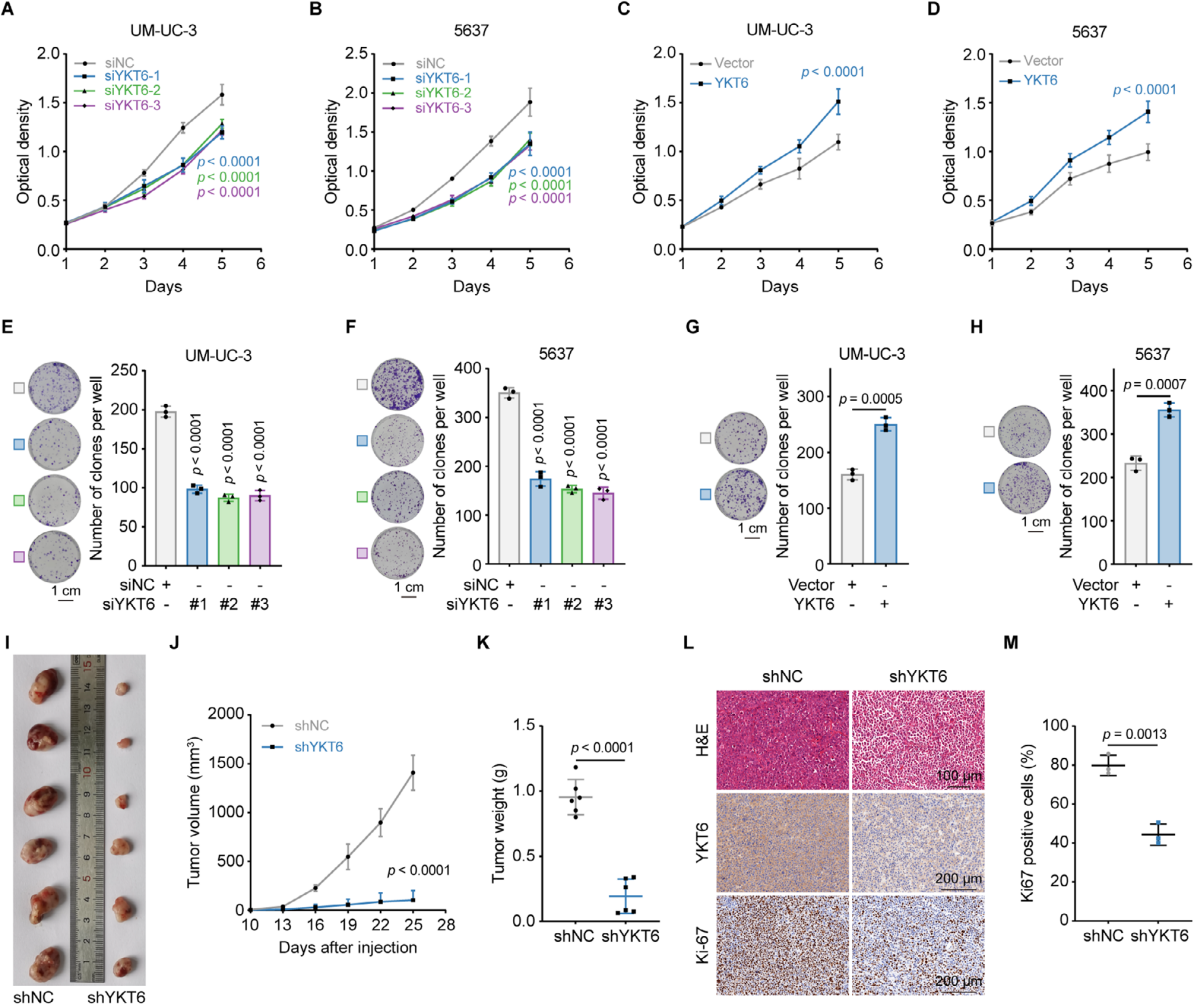
YKT6 knockdown suppresses BLCA proliferation in vitro and in vivo. A,B) The cell growth curve of siNC and siYKT6 in UM‐UC‐3 (A) and 5637 (B) cells was detected by MTT assay (n = 6 per group). C,D) MTT assay results of UM‐UC‐3 (C) and 5637 (D) with overexpressing YKT6 (n = 6 per group). E,F) Representative images and statistical chart of colony formation assays from the indicated groups with YKT6 knockdown in UM‐UC‐3 (E) and 5637 (F) cells (n = 3 per group). Scale bar: 1 cm. G,H) Representative images and statistical chart of colony formation assays from the indicated groups with YKT6 overexpression in UM‐UC‐3 (G) and 5637 (H) cells (n = 3 per group). Scale bar: 1 cm. I) Image of subcutaneous tumors in xenograft models of shNC‐transfected and shYKT6‐transfected UM‐UC‐3 cells. J) Tumor growth curve of xenograft models as indicated groups (n = 6 per group). K) Tumor weight of subcutaneous tumors in xenograft models (n = 6 per group). L) H&E and IHC staining of subcutaneous tumors in xenograft models. Scale bar: 100 and 200 µm. M) Statistical chart of Ki67 positive cells in shNC and shYKT6 group from xenograft model (n = 3 per group). Data are presented as mean values ± SD; one‐way ANOVA with Dunnett's multiple comparisons test at Day 5 (A,B); two‐tailed unpaired Student's t‐test at Day 5 (C,D); one‐way ANOVA with Dunnett's multiple comparisons test (E‐F); two‐tailed unpaired Student's t‐test (G‐H, K, M); two‐tailed unpaired Student's t‐test at Day 25 (J).

To evaluate the effects of YKT6 in BLCA progression, we first assessed the efficiency of siRNA, shRNA, and overexpression plasmids in altering YKT6 expression by qRT‐PCR and immunoblot assay (Figure S2A–I, Supporting Information). MTT assays indicated that YKT6 silencing significantly suppressed the proliferative capacity of UM‐UC‐3 and 5637 cells (**Figure** **2**A,B; Figure S2J,K, Supporting Information), whereas YKT6 overexpression enhanced this capacity (Figure 2C‐D). Consistent results were identified in colony formation assay (Figure 2E–H; Figure S2L,M, Supporting Information). For in vivo validation, stable knockdown of YKT6 in UM‐UC‐3 cells was established by lentiviral transfection. Subcutaneous implantation of shNC‐ and shYKT6‐transfected cells into nude mice revealed a markedly reduced volume and weight of tumors in the shYKT6 group in comparison with controls (Figure 2I–K). Immunohistochemistry (IHC) analysis further confirmed decreased Ki‐67‐positive cells in the shYKT6 group (Figure 2L,M), underscoring YKT6's role in BLCA growth regulation.

### 2.3 YKT6 Knockdown Induces G1 Phase Arrest in BLCA Cells

**Figure 3:**
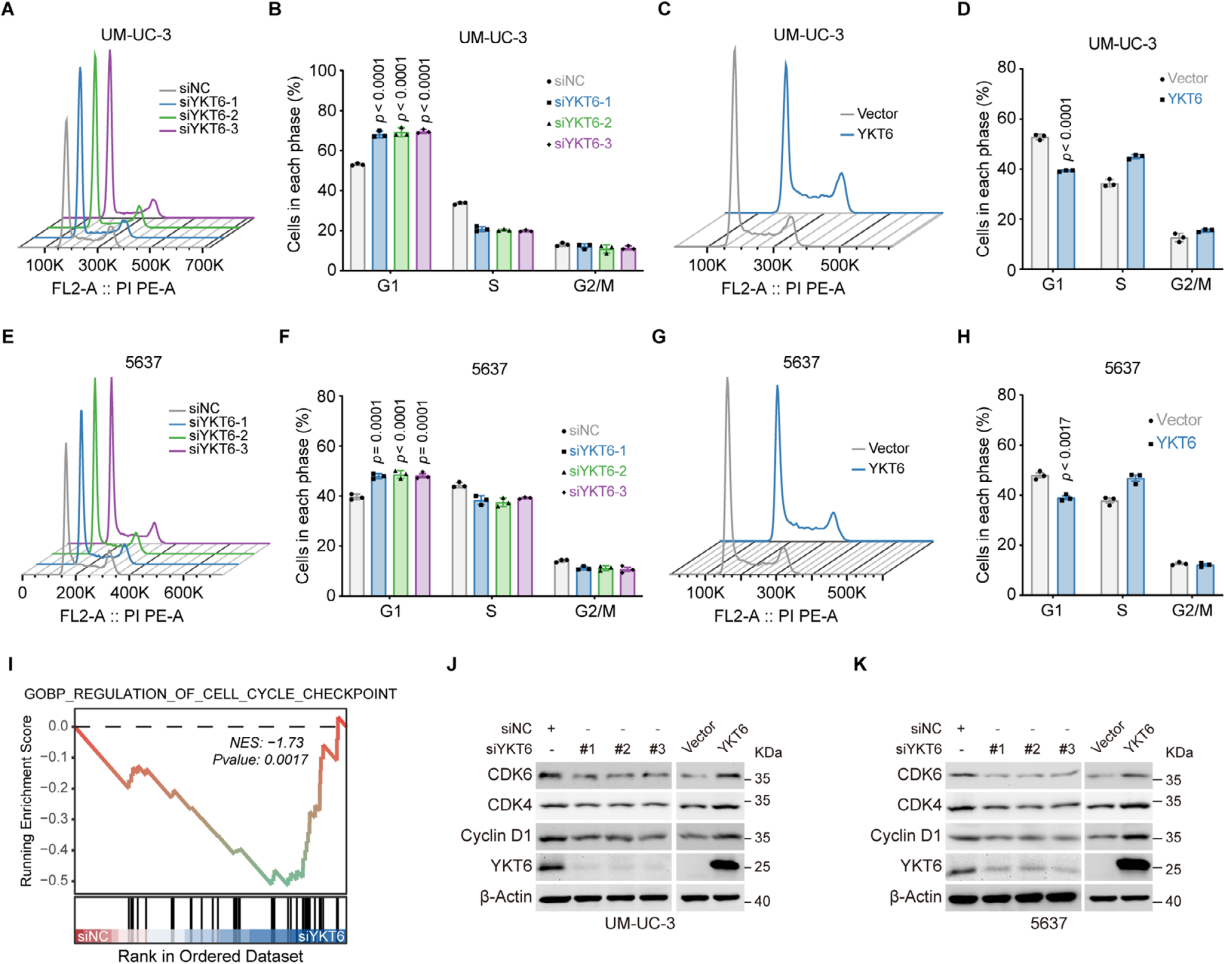
YKT6 knockdown suppresses cell cycle transition. A,B) Cell cycle distribution (A) and statistics (B, n = 3 per group) diagram of UM‐UC‐3 cells detected by flow cytometry as indicated groups. C,D) Cell cycle distribution (C) and statistics (D, n = 3 per group) diagram of UM‐UC‐3 cells detected by flow cytometry upon YKT6 overexpression. E,F) Cell cycle distribution (E) and statistic (F, n = 3 per group) diagram of 5637 cells detected by flow cytometry as indicated groups. G,H) Cell cycle distribution (G) and statistic (H, n = 3 per group) diagram of 5637 cells detected by flow cytometry upon YKT6 expression. I) GSEA of RNA‐seq data from YKT6‐silenced cells, highlighting enrichment in cell cycle checkpoint pathways. J,K) Immunoblot analysis of cell cycle‐related proteins in UM‐UC‐3 (J) and 5637 (K) cells upon YKT6 alteration. Data are presented as mean values ± SD; one‐way ANOVA with Dunnett's multiple comparisons test (B,F); two‐tailed unpaired Student's t‐test (D,H).

To assess the role of YKT6 in cell cycle regulation, flow cytometry assays were performed in BLCA cell lines. The results indicated that UM‐UC‐3 and 5637 cells were arrested at G1 phase upon YKT6 knockdown (**Figure** **3**A,B and E,F), whereas YKT6 overexpression promoted cell cycle transition (Figure 3C,D and G,H). Gene set enrichment analysis (GSEA) of RNA‐seq data further supported YKT6's association with the regulation of the cell cycle checkpoint (Figure 3I). Subsequently, we examined the cell cycle‐related proteins to explain the discovery above. Immunoblot assays showed that YKT6 knockdown downregulated key G1/S transition mediators, including CDK4, CDK6, and Cyclin D1, whereas YKT6 overexpression upregulated their expression (Figure 3J,K). These findings mechanistically link YKT6 to cell cycle dysregulation in BLCA.

### 2.4 YKT6 Enhances BLCA Metastasis In Vitro and In Vivo

**Figure 4:**
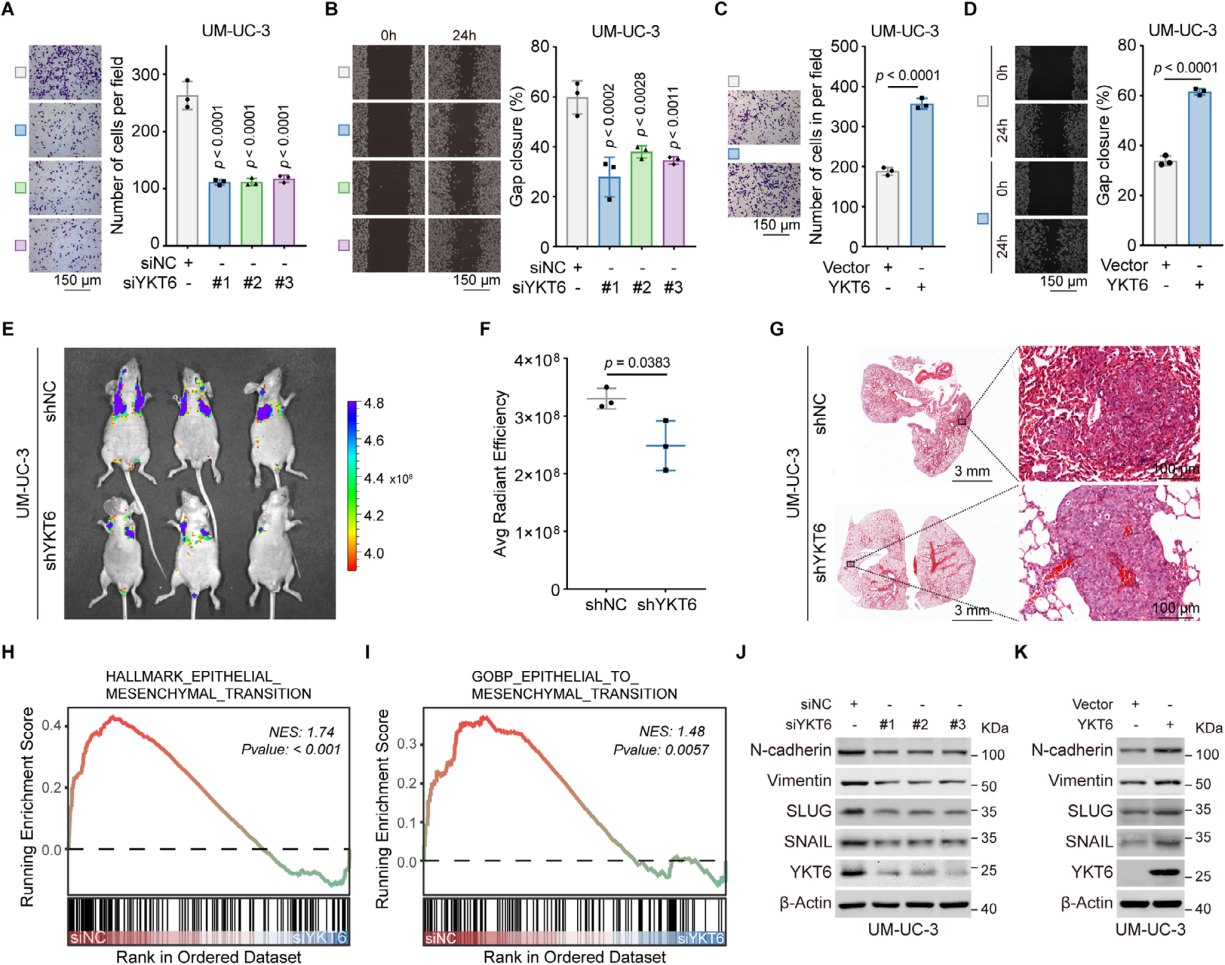
YKT6 enhances BLCA metastasis in vitro and in vivo. A,B) Transwell migration assay (A, n = 3 per group) and wound healing assay (B, n = 3 per group), representative images, and statistical diagram of UM‐UC‐3 cells upon YKT6 knockdown. Scale bar: 150 µm. C,D) Transwell migration assay (C, n = 3 per group) and wound healing assay (D, n = 3 per group) results of UM‐UC‐3 cells upon YKT6 overexpression. Scale bar: 150 µm. E) Lung fluorescence imaging of BALB/c‐nude mouse metastasis model injected with shNC and shYKT6 UM‐UC‐3 cells. F) Statistic plot of average radiant efficiency related to lung metastasis fluorescence (n = 3 per group). G) H&E staining of lung metastasis. Scale bar: 3 mm and 100 µm. H,I) GSEA of RNA‐seq data from YKT6‐silenced cells, showing significant enrichment of hallmark epithelial‐mesenchymal transition (H) and EMT‐related gene ontology terms (I). J,K) Immunoblot analysis of EMT‐related proteins upon YKT6 knockdown (J) or overexpression (K). Data are presented as mean values ± SD; one‐way ANOVA with Dunnett's multiple comparisons test (A,B); two‐tailed unpaired Student's t‐test (C,D,F).

The impact of YKT6 on BLCA cell migration was assessed using transwell migration and wound healing assays. YKT6 knockdown significantly suppressed migration in UM‐UC‐3 and 5637 cells (**Figure** **4**A,B; Figure S3A–C,E, Supporting Information), whereas YKT6 overexpression enhanced migratory capacity (Figure 4C,D; Figure S3D,F, Supporting Information). To evaluate metastatic potential in vivo, an experimental tumor lung metastasis model was constructed via tail vein injection of shNC‐ or shYKT6‐transfected UM‐UC‐3 cells. Mice in the shYKT6 group exhibited reduced lung fluorescence intensity compared to controls (Figure 4E,F), with hematoxylin and eosin (H&E) staining corroborating fewer metastatic nodules (Figure 4G). GSEA of RNA‐seq data from YKT6‐depleted cells uncovered a strong association between YKT6 and epithelial‐mesenchymal transition (EMT) signaling (Figure 4H,I). Immunoblot analysis confirmed that YKT6 knockdown downregulated N‐cadherin, Vimentin, SLUG, and SNAIL, whereas YKT6 overexpression induced the opposite trend (Figure 4J,K; Figure S3G,H, Supporting Information). These findings establish YKT6 as a driver of EMT‐mediated metastasis in BLCA.

### 2.5 YKT6 Activates Wnt/β‐Catenin Signaling in BLCA

**Figure 5:**
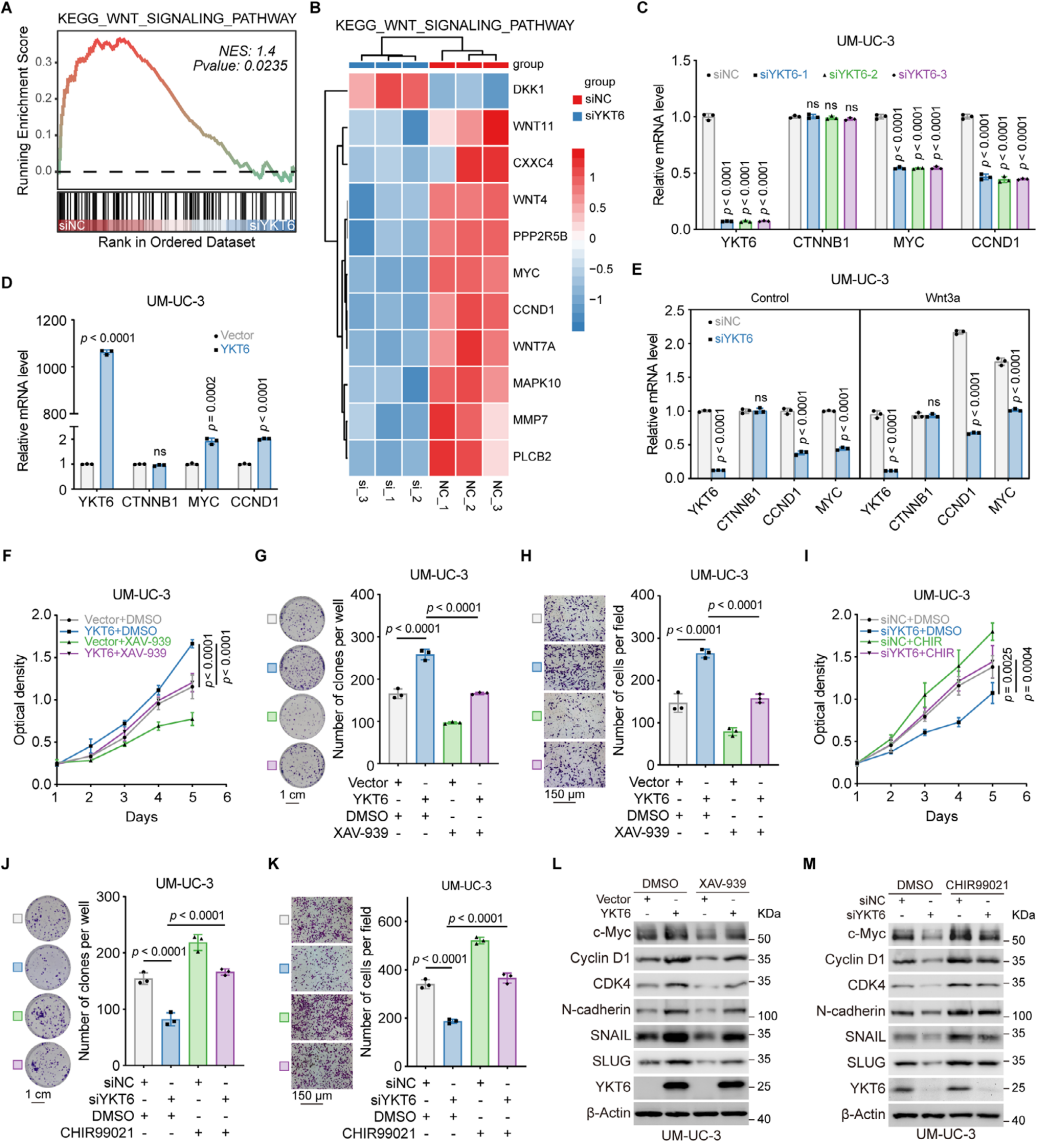
YKT6 correlates with Wnt/β‐catenin signaling activity in BLCA. A) GSEA of RNA‐seq data from YKT6‐silenced cells, showing significant enrichment of the KEGG Wnt signaling pathway. B) Heatmap of differentially expressed genes (DEGs) within the KEGG Wnt signaling pathway. C,D) qRT‐PCR analysis of β‐catenin target genes (*MYC*, *CCND1*) in UM‐UC‐3 cells following YKT6 knockdown (C, n = 3 per group) or overexpression (D, n = 3 per group). E) Rescue of YKT6 knockdown‐induced suppression of *MYC* and *CCND1* by Wnt3a treatment (100 ng mL^−1^) in UM‐UC‐3 cells (n = 3 per group). F–H) Pharmacological inhibition of Wnt signaling with XAV‐939 (10 µm) reverses YKT6‐overexpression‐driven proliferation (F, n = 6 per group), colony formation (G, n = 3 per group, Scale bar: 1 cm), and migration (H, n = 3 per group, Scale bar: 150 µm) in UM‐UC‐3 cells. I–K) Activation of Wnt signaling with CHIR99021 (4 µm) restores proliferation (I, n = 6 per group), colony formation (J, n = 3 per group, Scale bar: 1 cm), and migration (K, n = 3 per group, Scale bar: 150 µm) in YKT6‐silenced UM‐UC‐3 cells. L,M) Immunoblot analysis demonstrates that XAV‐939 (L) and CHIR99021 (M) counteract YKT6‐mediated changes in downstream targets. Data are presented as mean values ± SD; two‐tailed unpaired Student's t‐test (D,E); one‐way ANOVA with Dunnett's multiple comparisons test (C); one‐way ANOVA with Sidak's multiple comparisons test at Day 5 (F,I); one‐way ANOVA with Sidak's multiple comparisons test (G,H, J,K).

To uncover the underlying mechanism by which YKT6 promotes BLCA tumor growth and metastasis, RNA‐seq analysis was performed on 5637 cells transfected with siNC or siYKT6. GSEA results indicated that YKT6 knockdown caused reduced Wnt/β‐catenin signaling activity (**Figure** **5**A,B; Figure S4A, Supporting Information). Similarly, gene set variation analysis (GSVA) revealed a strong association between YKT6 expression and Wnt pathway activation (Figure S4B, Supporting Information). In addition, qRT‐PCR confirmed that YKT6 knockdown downregulated key downstream targets of Wnt/β‐catenin signaling, involving *MYC* and *CCND1*, while YKT6 overexpression upregulated their mRNA levels (Figure 5C–E and Figure S4C–E, Supporting Information). To functionally link YKT6 to Wnt/β‐catenin signaling, we modulated pathway activity using the specific activator CHIR99021 and inhibitor XAV‐939 in BLCA cells. As shown in MTT, colony formation, and transwell assay results, XAV‐939 rescued the enhanced proliferation and migration of YKT6‐overexpressing cells, while CHIR99021 obviously reversed the suppression caused by YKT6 silencing (Figure 5F–K; Figure S4F–K, Supporting Information). In addition, immunoblot assay further showed that YKT6‐mediated changes in Wnt/β‐catenin‐related proteins were recovered by XAV‐939 or CHIR99021, respectively (Figure 5L,M; Figure S4L,M, Supporting Information). Collectively, these results establish YKT6 as a crucial modulator of Wnt/β‐catenin signaling in BLCA.

### 2.6 YKT6 Enhances β‐Catenin Stability

**Figure 6:**
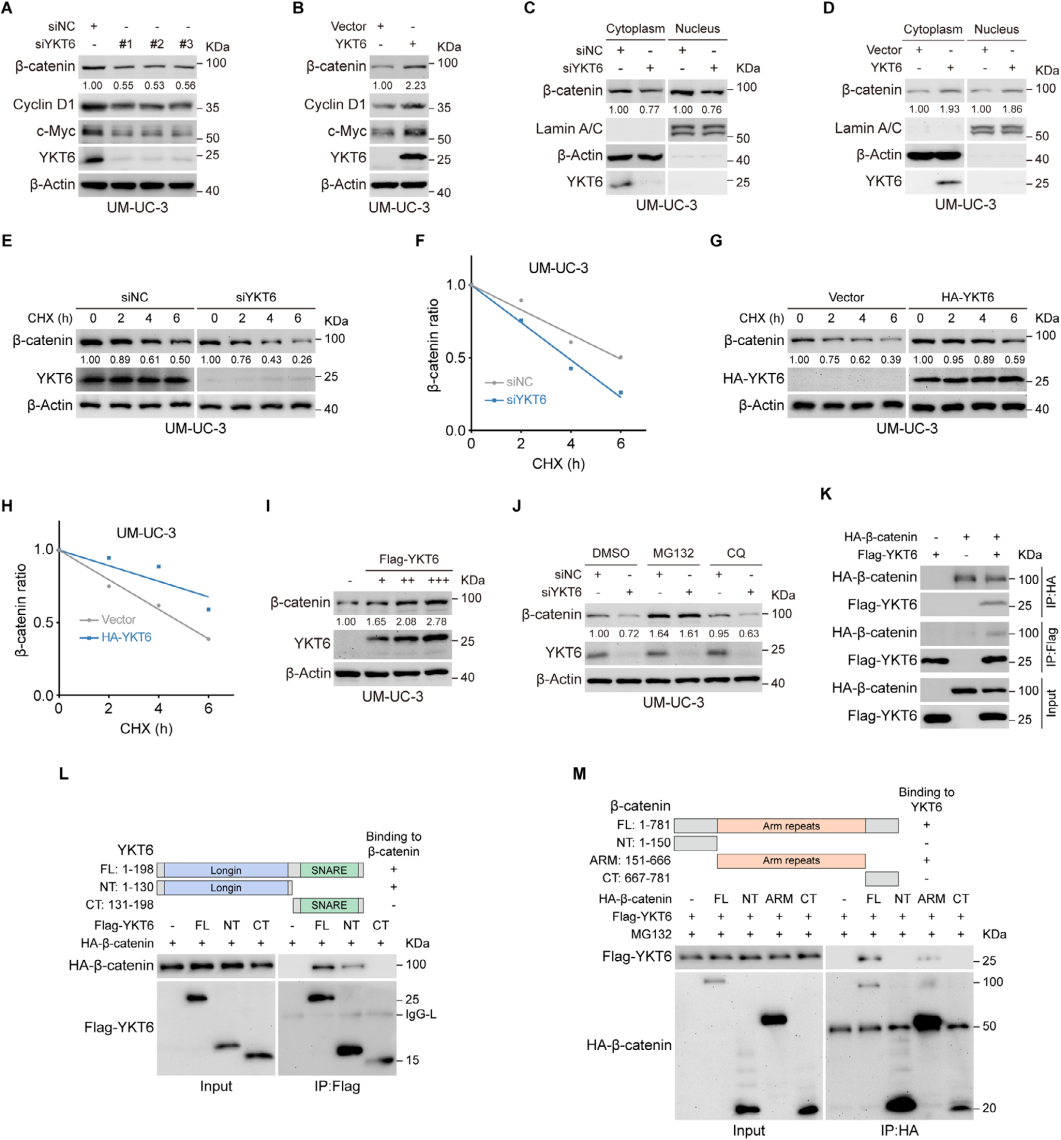
YKT6 enhances β‐catenin stability. A,B) Immunoblot analysis of β‐catenin and downstream proteins in UM‐UC‐3 cells following YKT6 knockdown (A) or overexpression (B). C,D) Cytoplasmic and nuclear β‐catenin levels in UM‐UC‐3 cells with YKT6 knockdown (C) or overexpression (D), analyzed by subcellular fractionation and Western blotting. Lamin A/C and β‐Actin served as nuclear and cytoplasmic markers, respectively. E,F) Cycloheximide (CHX, 50 µg mL^−1^) chase assay demonstrating accelerated β‐catenin degradation in YKT6‐silenced UM‐UC‐3 cells (E), quantified in (F). G,H) Delayed β‐catenin degradation in YKT6‐overexpressing UM‐UC‐3 cells (G) and corresponding quantification (H). I) Dose‐dependent increase in β‐catenin protein levels upon graded YKT6 overexpression. J) Alteration of β‐catenin protein abundance after YKT6 knockdown with MG132 (10 µm) or CQ (100 µm) treatment in UM‐UC‐3 cells. K) The interaction between YKT6 and β‐catenin in 293T cells was detected by exogenous co‐IP assay. L) Co‐IP analysis between HA‐β‐catenin and full‐length or truncations of Flag‐YKT6. The top panel is the schematic diagram of the truncations of YKT6. M) Co‐IP analysis between Flag‐YKT6 and full‐length or truncations of HA‐β‐catenin. The top panel is the schematic diagram of the truncations of β‐catenin.

The canonical Wnt/β‐catenin pathway functions as a critical driver of tumorigenesis, with β‐catenin serving as its central effector. To determine whether YKT6 modulates β‐catenin, we examined its expression in BLCA cells following YKT6 knockdown or overexpression. As immunoblot assays showed, YKT6 knockdown suppressed β‐catenin and downstream target expression (**Figure** **6**A; Figure S5A, Supporting Information), whereas YKT6 overexpression upregulated their expression (Figure 6B; Figure S5B, Supporting Information). Subcellular fractionation and immunoblot assay revealed that YKT6 knockdown reduced cytoplasmic and nuclear β‐catenin levels, while YKT6 overexpression elevated it (Figure 6C,D; Figure S5C,D, Supporting Information). Cycloheximide (CHX) assay showed that YKT6 knockdown increased the rate of β‐catenin degradation in UM‐UC‐3 and 5637 cells (Figure 6E,F; Figure S5E,F, Supporting Information), whereas YKT6 overexpression prolonged β‐catenin's half‐life (Figure 6G,H; Figure S5G,H, Supporting Information). Additionally, β‐catenin protein level was increased by YKT6 in a dose‐dependent manner (Figure 6I; Figure S5I, Supporting Information). Mechanistically, inhibition of proteasomal degradation with MG132 (but not autophagy inhibition by chloroquine (CQ)) restored β‐catenin level in YKT6‐silencing cells (Figure 6J; Figure S5J, Supporting Information). Furthermore, YKT6 silencing increased β‐catenin polyubiquitination, whereas YKT6 overexpression suppressed it (Figure S5K,L, Supporting Information). To probe the latent mechanism for observed β‐catenin changes, co‐IP assay and GST pull‐down assay were performed. This analysis result showed that there existed a binding between YKT6 and β‐catenin (Figure 6K; Figure S5M, Supporting Information). To identify the binding region between YKT6 and β‐catenin, truncations of both were constructed. Co‐IP assay revealed that the N‐terminal of YKT6 interacts with the Arm repeats domain of β‐catenin (Figure 6L,M). Additionally, immunofluorescence results showed that YKT6 and β‐catenin were colocalized in the cytoplasm of UM‐UC‐3 (Figure S5O, Supporting Information). Collectively, these findings supported the notion that YKT6 could maintain β‐catenin stability by inhibiting ubiquitination‐mediated proteasomal degradation.

### 2.7 YKT6 Stabilizes β‐Catenin Through USP7

**Figure 7:**
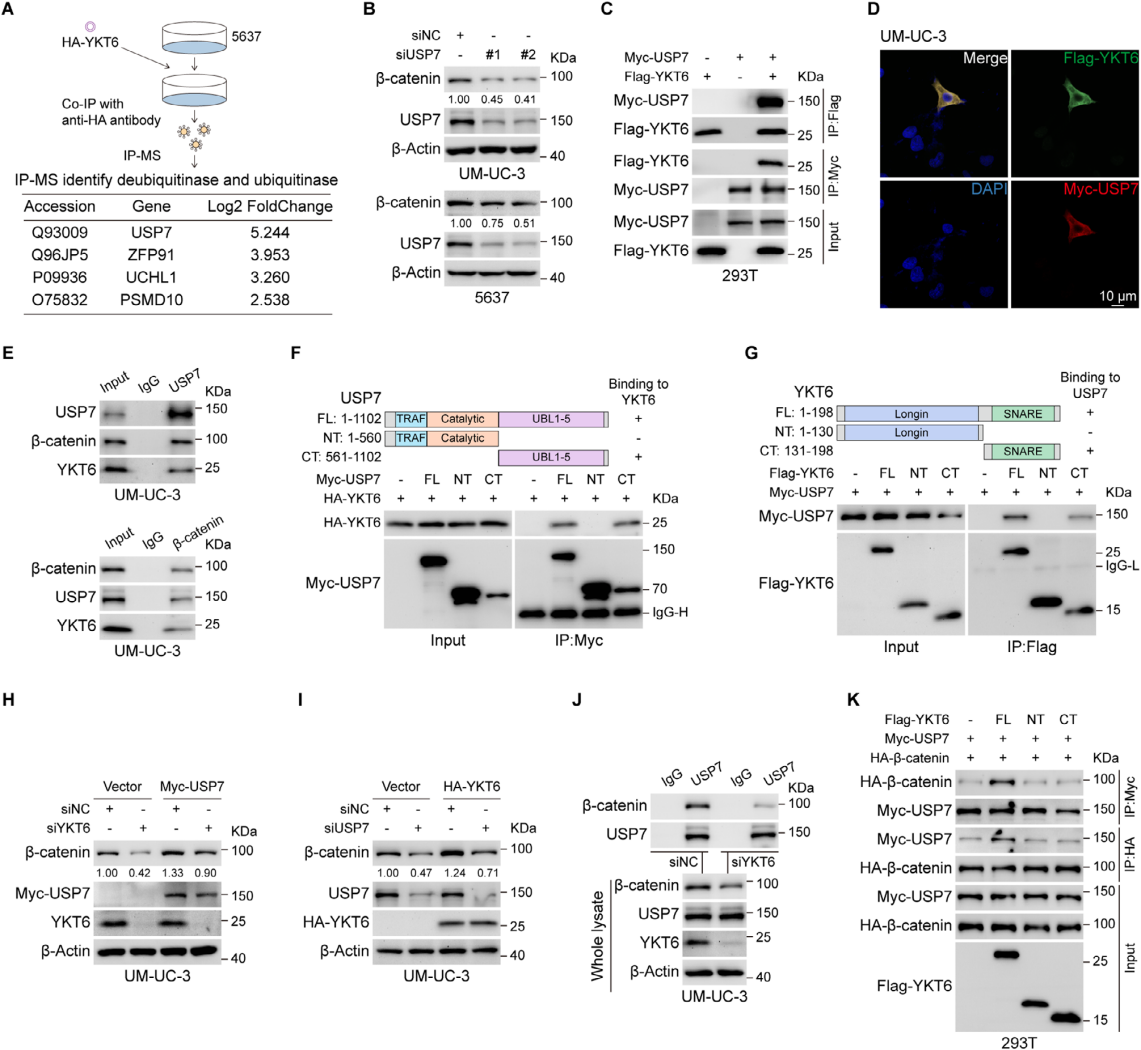
YKT6 stabilizes β‐catenin via USP7. A) Identification of YKT6‐interacting deubiquitinates and ubiquitinates by IP‐MS in HA‐tagged YKT6‐overexpressing 5637 cells. USP7 emerged as the top candidate. B) β‐catenin protein levels upon USP7 knockdown in UM‐UC‐3 and 5637 cells. C) The interaction between Flag‐YKT6 and Myc‐USP7 was examined by co‐IP assay in 293T cells. D) Immunofluorescence analysis of the co‐localization of Flag‐YKT6 and Myc‐USP7 in UM‐UC‐3. Scale bar: 10 µm. E) The endogenous co‐IP assay examined the interaction among YKT6, USP7, and β‐catenin in UM‐UC‐3 cells. F) The interaction between HA‐YKT6 and full‐length or truncations of Myc‐USP7 was detected by immunoblot assay. G) The interaction between Myc‐USP7 and full‐length or truncations of Flag‐YKT6 was detected by immunoblot assay. H) The β‐catenin protein level after YKT6 knockdown with or without USP7 overexpression in UM‐UC‐3. I) The β‐catenin protein level after USP7 knockdown with or without YKT6 overexpression in UM‐UC‐3. J) The interaction between USP7 and β‐catenin upon YKT6 knockdown was detected by endogenous co‐IP assay in UM‐UC‐3. K) The interaction between USP7 and β‐catenin upon full‐length or truncations of YKT6 overexpression was examined by immunoblot assay.

Although YKT6 lacks intrinsic enzymatic activity for protein ubiquitination regulation, we hypothesized that it might modulate β‐catenin stability via regulating ubiquitination‐associated enzymes. To uncover this, we performed immunoprecipitation‐mass spectrometry (IP‐MS) in YKT6‐overexpressing 5637, identifying USP7, ZFP91, UCHL1, and PSMD10 as candidates (**Figure** **7**A). Immunoblot analysis indicated that USP7 knockdown downregulated β‐catenin in BLCA cell lines, whereas knockdown of ZFP91, UCHL1, and PSMD10 did not (Figure 7B; Figure S6A, Supporting Information). Based on this, we focused on the role of USP7 in YKT6‐mediated regulation of β‐catenin. Subsequently, co‐IP and GST pull‐down assay demonstrated the binding between USP7 and YKT6 (Figure 7C; Figure S6B, Supporting Information). Immunofluorescence analysis indicated that there existed cytoplasmic colocalization between USP7 and YKT6 (Figure 7D). In addition, USP7 also bound to β‐catenin (Figure S6C,D, Supporting Information). Endogenous co‐IP identified the binding network among YKT6, USP7, and β‐catenin in BLCA cell lines (Figure 7E; Figure S6E, Supporting Information). Further co‐IP analysis revealed that C‐terminal of YKT6 interacts with the C‐terminal of USP7, and the N‐terminal of β‐catenin interacts with the N‐terminal of USP7 (Figure 7F,G; Figure S6F,G, Supporting Information). In addition, USP7 overexpression rescued β‐catenin levels in YKT6‐depleted cells (Figure 7H; Figure S6H, Supporting Information), whereas YKT6 partially restored β‐catenin in USP7‐silenced cells (Figure 7I; Figure S6I, Supporting Information). YKT6 knockdown markedly impaired USP7‐β‐catenin interaction in BLCA cell lines (Figure 7J; Figure S6J, Supporting Information). In the meanwhile, YKT6 overexpression promoted the interaction between β‐catenin and USP7 (Figure S6K, Supporting Information). Additionally, co‐IP analysis indicated that only full‐length YKT6 could promote the interaction between USP7 and β‐catenin, whereas truncations of YKT6 could not (Figure 7K).

In addition, the immunoblot and qRT‐PCR results showed that overexpressing USP7 could upregulate β‐catenin protein level but not the mRNA level (Figure S7A–D, Supporting Information). β‐catenin overexpression could not influence both the protein and mRNA levels of YKT6 and USP7 (Figure S7E–H, Supporting Information). Collectively, these results demonstrate that full‐length YKT6 recruits USP7 to β‐catenin, thereby promoting its stabilization, with YKT6 functioning as a scaffold in this regulatory process.

### 2.8 YKT6 Reduces β‐Catenin Polyubiquitination Mediated by β‐TrCP Through USP7

**Figure 8:**
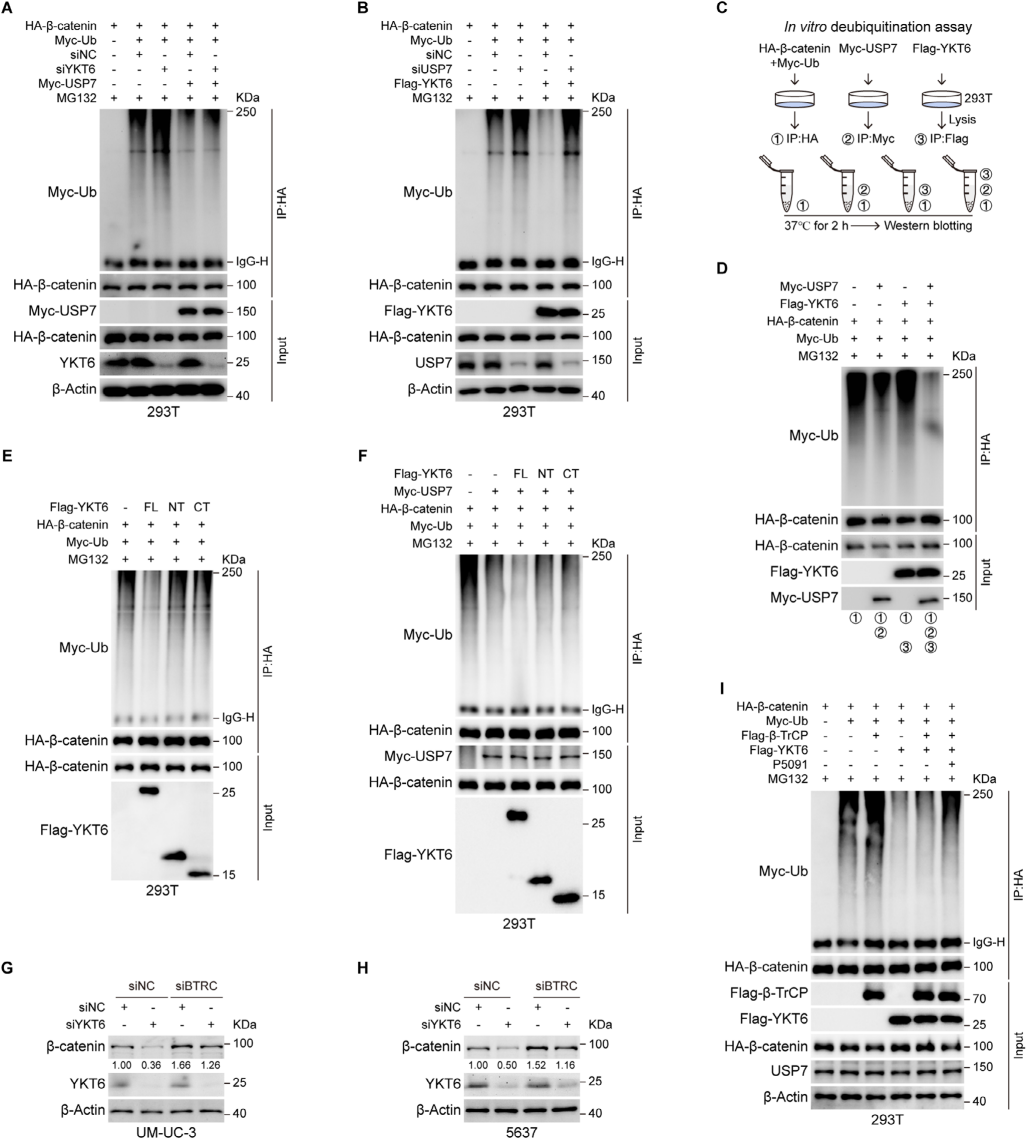
YKT6 reduces β‐catenin polyubiquitination through USP7. A,B) Ubiquitination assay in 293T cells demonstrated that USP7 (A) or YKT6 overexpression (B) reduces β‐catenin polyubiquitination under proteasome inhibition (MG132, 10 µm). C) Schematic diagram of the in vitro deubiquitination assay. D) Polyubiquitination of β‐catenin was detected by immunoblot assay upon USP7 or YKT6 overexpression from in vitro deubiquitination assay. E) Ubiquitination of β‐catenin upon overexpressing full‐length or truncations of Flag‐YKT6 in 293T cells. F) The effects of full‐length or truncated YKT6 on ubiquitination of β‐catenin in the presence of USP7. G,H) Immunoblot analysis of β‐catenin protein level upon knockdown of YKT6 and BTRC in UM‐UC‐3 and 5637 cells. I) The ubiquitination of β‐catenin mediated by β‐TrCP, YKT6, and P5091 (10 µM) was detected by immunoblot assay.

To better understand the function of USP7 in the alteration of β‐catenin modulated by YKT6, an ubiquitination assay was carried out. The result indicated that YKT6 knockdown increased the polyubiquitination of β‐catenin, an effect that was reversed by ectopic USP7 expression (**Figure** **8**A). At the same time, USP7 knockdown increased the ubiquitination of β‐catenin, while subsequent ectopic expression of YKT6 did not significantly affect this enhancement (Figure 8B). In vitro deubiquitination assay demonstrated that YKT6 enhanced the deubiquitination activity of USP7 on β‐catenin (Figure 8C,D). Further results from the ubiquitination assay revealed that only full‐length YKT6 could decrease the polyubiquitination of β‐catenin (Figure 8E). Since only full‐length YKT6 can promote the interaction between USP7 and β‐catenin, we further investigated whether full‐length YKT6 is also required for USP7‐mediated deubiquitination of β‐catenin. The result confirmed that only full‐length YKT6 could promote the deubiquitination of β‐catenin modulated by USP7 (Figure 8F). To investigate the specific type of ubiquitin chain involved, ubiquitin mutant plasmids were constructed. The result from the ubiquitination assay indicated that YKT6 and USP7 mainly removed the K48‐linked ubiquitin chains from β‐catenin (Figure S8A,B, Supporting Information).

Based on our previous finding that E3 ubiquitin‐protein ligase ZFP91 does not regulate β‐catenin protein stability (Figure S6A, Supporting Information), we further investigated whether YKT6 influences β‐catenin ubiquitination by E3 ligases other than through its role in mediating USP7‐driven deubiquitination. To this end, we focused on β‐TrCP, a canonical E3 ligase known to target β‐catenin.^[^ ^29^, ^30^ ^]^ The results indicated that knockdown of β‐TrCP significantly increased β‐catenin protein levels, but this effect was partially attenuated by further knockdown of YKT6 (Figure 8G,H; Figure S8C,D, Supporting Information). Further co‐IP analysis showed no evidence of an interaction between YKT6 and β‐TrCP. (Figure S8E, Supporting Information). Subsequently, the ubiquitination assay result revealed that overexpressing YKT6 could reduce the polyubiquitination of β‐catenin attached by β‐TrCP (Figure S8F, Supporting Information). In addition, P5091 (USP7 inhibitor) suppressed YKT6‐mediated reduction of β‐TrCP‐induced ubiquitination of β‐catenin (Figure 8I). Altogether, these findings demonstrated that YKT6 reduces β‐catenin polyubiquitination mediated by β‐TrCP through USP7.

### 2.9 YKT6‐USP7‐β‐Catenin Axis Promotes BLCA Progression

**Figure 9:**
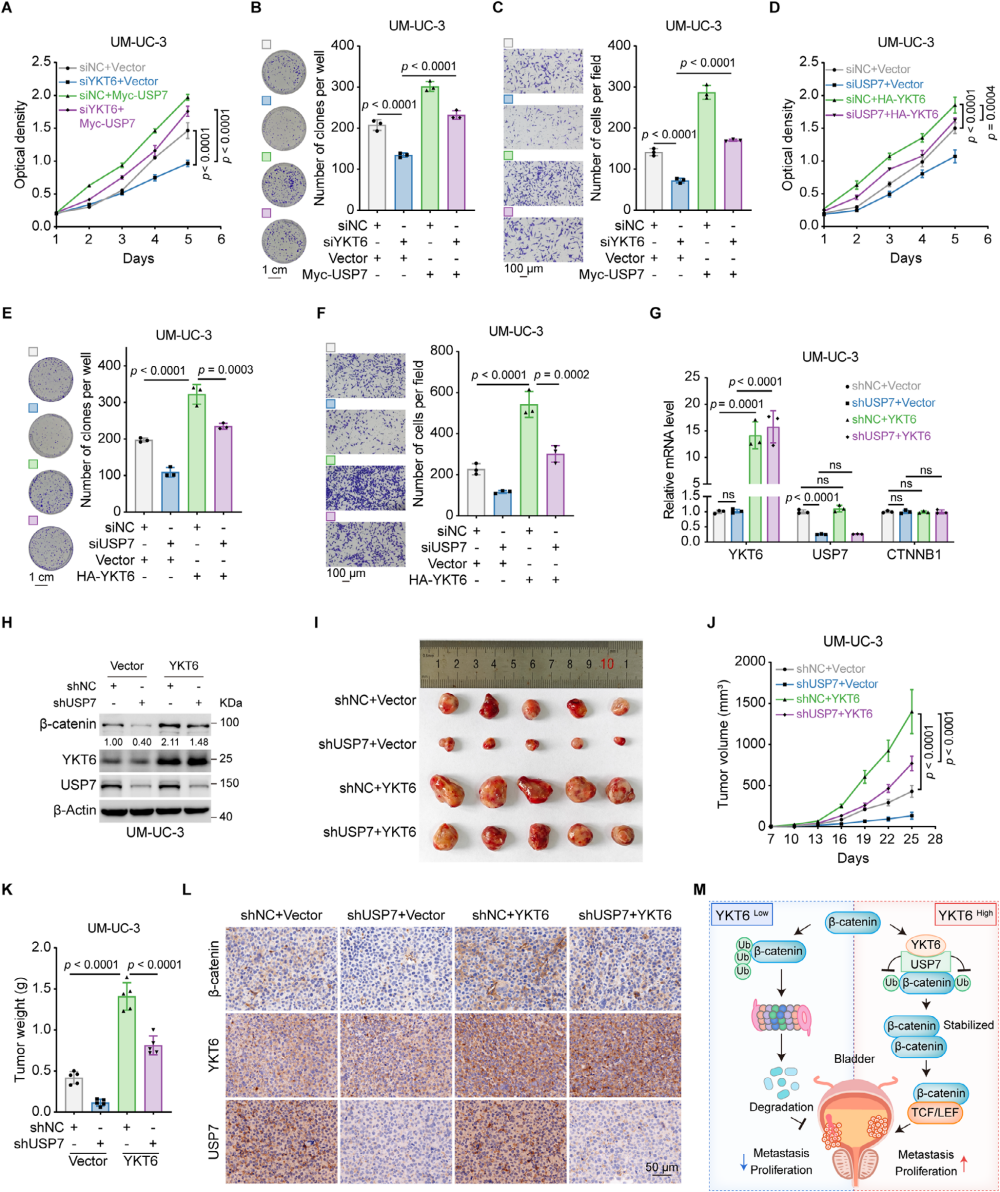
YKT6‐USP7‐β‐catenin axis promotes BLCA progression. A–C) Overexpression of USP7 reverses YKT6‐knockdown‐driven proliferation (A, n = 6 per group), colony formation (B, n = 3 per group, Scale bar: 1 cm), and migration (C, n = 3 per group, Scale bar: 100 µm) in UM‐UC‐3 cells. D–F) Knockdown of USP7 impairs YKT6‐overexpression‐driven proliferation (D, n = 6 per group), colony formation (E, n = 3 per group, Scale bar: 1 cm), and migration (F, n = 3 per group, Scale bar: 100 µm) in UM‐UC‐3 cells. G,H) The mRNA (G, n = 3 per group) and protein (H) levels of YKT6, USP7, and β‐catenin upon USP7 knockdown or YKT6 overexpression. I) Image of subcutaneous tumors in xenograft models of indicated UM‐UC‐3 cells. J) Tumor growth curves of xenograft models as indicated groups (n = 5 per group). K) Tumor weight of subcutaneous tumors in xenograft models (n = 5 per group). L) IHC staining of β‐catenin, YKT6, and USP7 in subcutaneous tumors from the xenograft model. M) The mechanistic diagram of this study. YKT6 interacts with USP7 to deubiquitinate and stabilize β‐catenin. Accumulative β‐catenin enters the nucleus and directs target gene expression through TCF/LEF, promoting BLCA progression. Data are presented as mean values ± SD; one‐way ANOVA with Sidak's multiple comparisons test at Day 5 (A,D); one‐way ANOVA with Sidak's multiple comparisons test (B,C, E–G,K); one‐way ANOVA with Sidak's multiple comparisons test at Day 25 (J).

To further understand the role of the YKT6‐USP7‐β‐catenin regulatory axis in BLCA, we performed both in vitro and in vivo experiments. As shown in MTT, colony formation, and transwell assay results, overexpression of USP7 rescued the reduced proliferation and migration of YKT6‐knockdown cells (**Figure** **9**A–C; Figure S9A–C, Supporting Information). In addition, USP7 knockdown obviously suppressed the promotion caused by YKT6 overexpression (Figure 9D–F; Figure S9D–F, Supporting Information). Subsequently, stable overexpression of YKT6 and knockdown of USP7 in UM‐UC‐3 cells were constructed by lentivirus transfection with efficiency validation (Figure 9G,H). In the xenograft model, overexpression of YKT6 upregulated the tumor growth and weight, and these effects were reversed by P5091 or USP7 knockdown (Figure 9I–K; Figure S9G–I, Supporting Information). IHC analysis demonstrated that overexpressing YKT6 upregulated the abundance of β‐catenin, which was reversed by USP7 inhibition (Figure 9L; Figure S9J, Supporting Information). In conclusion, YKT6‐USP7‐β‐catenin regulatory axis promotes BLCA progression in vivo and in vitro.

## 3 Discussion

SNARE proteins, localized to organelles and cell membranes, modulate exosome biogenesis and release with precision.^[^ ^31^, ^32^ ^]^ Recent research indicates that SNARE family members are closely associated with various tumor progression and chemoresistance.^[^ ^33^, ^34^, ^35^, ^36^, ^37^, ^38^, ^39^ ^]^ Within this family, YKT6 mediates both carcinogenesis and development. Related studies indicate that, YKT6 is significantly upregulated in hepatocellular carcinoma, pancreatic cancer, oral squamous cell carcinoma, and NSCLC.^[^ ^13^, ^17^, ^18^, ^40^ ^]^ In pancreatic cancer and NSCLC, YKT6 affects tumor progression by participating in the formation or excretion of exosomes in tumor cells.^[^ ^14^, ^18^ ^]^ Consistent with previous research, our present analysis confirms that YKT6 expression exhibits upregulation in BLCA tissues and cell lines compared with adjacent normal tissues and urothelial cells. Additionally, YKT6 expression level in high‐grade is higher than low‐grade and predicts poor prognosis from larger public datasets and tissue microarray. Furthermore, functional studies using gain and loss‐of‐function models establish YKT6's role in driving BLCA proliferation and metastasis both in vitro and in vivo settings.

Dysregulated Wnt/β‐catenin signaling is intricately linked to diverse facets of cancer, encompassing initiation, progression, malignant transformation, and more.^[^ ^41^, ^42^ ^]^ Upon activation of upstream signals, β‐catenin translocates into the nucleus as the central effector, where it modulates gene expression, thereby contributing to cell proliferation, differentiation, stem cell maintenance, and so on.^[^ ^43^, ^44^ ^]^

The transformation of epithelial cells to mesenchymal cells, accompanied by molecular changes, can take place during the EMT. Within human malignancies, the EMT process enhances the capacity for cancer metastasis and drug resistance development. Among the various modulators of EMT, the Wnt/β‐catenin signaling axis has been identified as a multifunctional regulator.^[^ ^45^ ^]^ Moreover, multiple target genes, regulated by the Wnt/β‐catenin signaling, engage in facilitating cancer cell invasion, migration, and metastatic dissemination.^[^ ^46^, ^47^ ^]^ Cell division is stringently governed by numerous evolutionarily preserved cell cycle regulatory mechanisms, so as to ensure the creation of two genetically indistinguishable cells.^[^ ^48^ ^]^ Dysregulation of cell cycle progression serves as one of the essential mechanisms for oncogenesis, creating regulators of the cell cycle apparatus as reasonable anticancer therapeutic targets.^[^ ^49^ ^]^

The canonical Wnt/β‐catenin pathway has a crucial function in modulating tumorigenesis through halting the cell cycle at diverse phases. When β‐catenin is stabilized, it enters the nucleus and forms a transcriptional complex with TCF/LEF cofactors, thereby upregulating key cell cycle regulators such as CCND1 and MYC. These genes are critical for driving the G_1_/S phase transition, a pivotal checkpoint in cell cycle progression.^[^ ^25^, ^50^, ^51^ ^]^

Current literature supports an established role for YKT6 in the regulation of Wnt ligand secretion. Specifically, YKT6 has been shown to facilitate Wnt release via exosomes derived from multivesicular bodies in both Drosophila and human cells.^[^ ^52^ ^]^ Further studies indicate that Ykt6 mediates endosomal recycling, particularly through Rab4‐positive compartments, which is essential for the long‐range secretion and signaling of Wnt ligands.^[^ ^53^ ^]^ Additionally, phosphorylation of the Ykt6 SNARE domain modulates its membrane recruitment and subsequent secretion of Wnt proteins and Wnt‐containing extracellular vesicles.^[^ ^54^ ^]^ Consistent with these findings, depletion of Ykt6 results in intracellular accumulation of Wnt ligands and impaired Wnt signaling.^[^ ^52^, ^53^ ^]^ Thus, while YKT6 is a recognized regulator of Wnt ligand secretion, its potential involvement in downstream β‐catenin signaling, beyond ligand secretion, remains unclear and merits further investigation.

In this study, our findings are consistent with a model in which YKT6 modulates β‐catenin stability through a scaffolding mechanism that appears to operate independently of its established roles in membrane trafficking. The results demonstrated that YKT6 could modulate the activity of Wnt/β‐catenin signaling through its ability to stabilize β‐catenin protein. The critical mediator of the canonical Wnt/β‐catenin pathway is the cytoplasmic protein β‐catenin, which remains a versatile protein, exhibiting versatility in various cellular events and human diseases. The central regulatory paradigm of the Wnt/β‐catenin pathway hinges on a dynamic equilibrium between kinase‐directed phosphorylation events and proteasomal degradation mechanisms of β‐catenin.^[^ ^55^, ^56^ ^]^ Research has reported that several deubiquitinating enzymes, including USP8, USP9X, USP20, and USP33, modulate the β‐catenin deubiquitination, thereby driving growth, metastasis of cancer cells or other biological process.^[^ ^57^, ^58^, ^59^, ^60^ ^]^ Through IP‐MS screening, we identified USP7 as a critical mediator of YKT6‐dependent β‐catenin stabilization. This finding aligns with prior studies demonstrating USP7's role in modulating β‐catenin stability.^[^ ^61^, ^62^ ^]^

While our data point to a potential scaffolding function for YKT6 in oncogenic signaling, we acknowledge that our experiments do not address whether this role is independent of its canonical function in vesicle fusion. It is plausible that the observed oncogenic effects are a composite of both this novel scaffolding mechanism and its canonical role in secretion. Specifically, it would be valuable for future studies to determine if disrupting vesicle fusion impairs YKT6‐mediated stabilization of β‐catenin, which will be critical to confirm the functional independence of this non‐canonical pathway. Furthermore, while upregulation of YKT6 was detected in the ZNWU in‐house dataset, we acknowledge the limitations of our analysis due to the limited sample size (n = 36) and potential selection bias. The survival of cancer patients is influenced by multiple factors. The survival association between upregulated YKT6 expression and poor prognosis of BLCA patients could be influenced by confounding factors and requires further validation. In this study, we demonstrated that a USP7 inhibitor (P5091) could reverse YKT6‐driven tumor growth in mice, which suggests the axis is druggable at the level of USP7. However, SNARE proteins are known to be challenging drug targets, and YKT6 itself was not directly targeted by an inhibitor in our study. The clinical translation of these findings is a distant prospect that requires significant future work, including the development and optimization of specific YKT6 or USP7 inhibitors for clinical use in bladder cancer, along with rigorous evaluation of their in vivo toxicity and therapeutic index.

In conclusion, YKT6 is upregulated in BLCA progression and is tightly interrelated with unfavorable prognosis. Mechanistically, YKT6 could stabilize β‐catenin via USP7‐mediated deubiquitination, thereby potentiating Wnt/β‐catenin signaling activity (Figure 9M). Altogether, our study elucidates the role of the YKT6/USP7/β‐catenin axis and provides foundational insights that may inform future strategies for precision BLCA therapy.

## 4 Experimental Section

### Cell Lines and Reagents

Cell lines in the current study were acquired from the Cell Bank of the Chinese Academy of Sciences (Shanghai, China) with verification. UM‐UC‐3 and J82 were cultured in MEM medium. T24, 5637, SCaBER, and SV‐HUC‐1 were cultured in RPMI 1640 medium. HEK293T were cultured in DMEM medium. Culture media were complemented with 10% fetal bovine serum (FBS), and 1% penicillin‐streptomycin solution (Gibco, USA) and utilized to maintain cells at 37 °C with 5% CO_2_. Wnt agonist CHIR99021 (S1263) and recombinant human Wnt3a protein (HZ‐1296) were purchased from Selleck and Proteintech, respectively. P5091 (HY‐15667) and Wnt inhibitor XAV‐939 (HY‐15147) were obtained from MedChemExpress. CQ was acquired from Selleck (S6999), and MG132 was obtained from Selleck (S2619).

### Human Samples

This study received ethical approval from the Institutional Ethics Committee of Zhongnan Hospital, Wuhan University (approval number: 2021125), and informed consent was acquired from participants. BLCA tissues and adjacent normal tissues were gathered post‐radical cystectomy. Surgical specimens were processed by pathologists immediately after surgery. Both cancer and adjacent normal tissues were histologically confirmed.

### Tissue RNA Extraction

Pre‐cooled scissors and forceps were used to chop the tissue samples into appropriate sizes on dry ice, and the fragments were immediately transferred to a 1.5 mL RNase‐free EP tube containing Trizol reagent and sterile grinding beads. The tissue samples were homogenized thoroughly using a tissue homogenizer. After brief centrifugation at 4 °C for 30 secs, the Trizol‐based tissue homogenate was immediately transferred to a new 1.5 mL RNase‐free EP tube. Chloroform was added, followed by vortexing for 15 s to mix thoroughly. The mixture was then incubated at room temperature for 5 mins, and subsequently centrifuged at 13 300 g for 15 mins at 4 °C. The upper aqueous phase was carefully transferred to a new RNase‐free EP tube. After the addition of isopropanol, the solution was mixed thoroughly by gentle inversion. After incubation at room temperature for 10 mins, the sample was centrifuged at 13 300 g for 15 mins at 4 °C. Following centrifugation, the supernatant was discarded, and the RNA pellet was retained. The pellet was washed twice with 75% ethanol (prepared with RNase‐free water), briefly air‐dried, and then dissolved in an appropriate volume of DEPC‐treated water. Finally, RNA concentration was determined using a NanoDrop spectrophotometer.

### Quantitative Reverse Transcription PCR (qRT‐PCR)

Total RNA was isolated using the HiPure Total RNA Mini Kit (R4111‐03, Magen, China). The NanoDrop One instrument was used to evaluate the concentration and quality of extracted RNA. Subsequently, isolated RNA was applied to reverse transcription into cDNA, referring to the ReverTra Ace qPCR RT Kit (FSQ‐101, TOYOBO, Japan). Reaction system including iQ SYBR Green Supermix (1 725 125, Bio‐Rad, USA), validated primers, and template cDNA was applied to the StepOnePlus Real‐Time PCR System (Thermo Fisher, USA). Relative quantification depends on 2^−ΔΔCt^ method, with *ACTB* as the endogenous reference gene. All the primer sequences mentioned were provided in Table S2 (Supporting Information).

### Western Blot and Immunoprecipitation Analysis

Cells pretreated as indicated were harvested via centrifugation, followed by PBS washing. The lysis buffer, including RIPA, PMSF (1 mm), and phosphatase inhibitors, was added to the cell pellet and lysed for 30 mins on ice. Each group tube was vortexed vigorously every 10 mins. Then, the samples were denatured with 5× loading buffer at 100 °C for 10 mins. Proteins were resolved via SDS‐PAGE gel, following transferred onto a 0.45‐µm PVDF membrane. The membrane was subsequently subjected to blocking with 5% non‐fat milk in TBST buffer. Next, primary and secondary antibodies were incubated sequentially. Finally, immunoblots were visualized using the BioSpectrum 515 Imaging System (UVP). β‐Actin protein serves as an internal control to normalize protein loading.

For co‐immunoprecipitation (co‐IP), cells were harvested and lysed with a mixture including lysis buffer, PMSF (1 mm), and phosphatase inhibitor. After lysing for 1 hr at 4 °C, the lysate then was divided into input and immunoprecipitation groups and added specific antibody overnight in a rotary mixer. Suspension of washed magnetic beads were added to each sample, and incubated for 1.5 hrs at 4 °C, followed by gentle washing for three times. Removed the supernatant, samples including beads were boiled with 1× SDS‐PAGE loading buffer for 10 mins at 100 °C. Western blotting assay was conducted to test the target proteins.

Primary and secondary antibodies for Western blot (WB) and immunoprecipitation (IP) analysis used in this study were provided in Table S3 (Supporting Information).

### GST Pull‐Down Assay

Recombinant GST, GST‐YKT6, and His‐β‐catenin proteins were purchased from CUSABIO Co., Ltd. (Wuhan, China). For the binding assay, 2 µg of His‐β‐catenin was incubated with 2 µg of GST or GST‐YKT6 fusion protein in binding buffer supplemented with protease and phosphatase inhibitors for 4 hrs at 4 °C. The mixtures were then incubated with 20 µL of pre‐washed glutathione‐Sepharose beads for 2 hrs at 4 °C. After three washes with IP wash buffer, bound proteins were eluted with 1× loading buffer, denatured at 100 °C for 10 minsM, and analyzed by immunoblot assay.

### CHX‐Chase Assay

To evaluate the target protein half‐life, cells were treated with CHX at the indicated time (50 µg mL^−1^) in culture medium. Subsequently, cells were harvested for immunoblot analysis. The relative quantification of target protein levels was calculated using ImageJ software.

### IP‐MS Assay

5637 cells were transiently transfected with the HA‐tagged Vector or YKT6 plasmid for 48 hrs, after which IP was conducted. Magnetic beads were harvested, followed by protein denaturation, cysteine reduction, and alkylation in the reaction buffer. The eluates were diluted with H_2_O and digested with trypsin. Next, the pH was brought down to 6.0 by TFA to end this digestion. After centrifugation, the self‐made SDB desalting columns were utilized to purify the peptide, followed by vacuum drying and storage at ‐20 °C. Further mass spectrometry analysis was conducted on a trapped ion mobility quadrupole time‐of‐flight mass spectrometer timsTOF Pro (Bruker Daltonics, USA). An UltiMate 3000 RSLCnano system (Thermo Fisher Scientific, USA) was coupled online to the timsTOF Pro with a CaptiveSpray nano ion source (Bruker Daltonics, USA). MS raw data were processed using MaxQuant software (V2.0.1) with the Andromeda search engine. Spectra files were processed through the Uniprot human database.

### Ubiquitination Assay

Cells were treated as indicated. Eight hrs before harvesting cells, the cell culture was supplemented with MG132 (10 µM). After washing with PBS and centrifugation, cell pellets were lysed in RIPA buffer containing PMSF (1 mm) and phosphatase inhibitors. The following steps were in accordance with co‐IP.

### In Vitro Deubiquitination Assay

The deubiquitination assay was performed according to a previously published study.^[^ ^63^ ^]^ HA‐β‐catenin and Myc‐Ub plasmids were transfected into 293T cells and treated with 10 µm MG132 8 h before cell collection. Flag‐YKT6 and Myc‐USP7 proteins were immunoprecipitated using anti‐Flag and anti‐Myc antibodies (listed in Table S3, Supporting Information), respectively. YKT6 and USP7 proteins were then eluted under non‐denaturing conditions. Polyubiquitinated β‐catenin was incubated with either YKT6 or USP7 in a buffer containing 50 mM Tris‐HCl, 5 mm MgCl_2_, 2 mm DTT, 2 mm ATP‐Na_2_, and proteasome inhibitors at 37 °C for 2 hrs. The reaction was terminated by adding 5× loading buffer, followed by denaturation at 100 °C for 10 mins. Finally, the ubiquitination levels were analyzed by immunoblot analysis.

### RNA Sequencing and Bioinformatic Analysis

5637 cells were transfected with siNC and siYKT6 for 48 hrs. Total RNA was isolated, and its quality was subsequently evaluated. The processing of 2 µg of high‐quality RNA was carried out using the KCTM Stranded mRNA Library Prep Kit for Illumina (DR08402; Wuhan Seqhealth Co., Ltd.) according to the manufacturer's protocol. PCR products were size‐selected, quantified, and sequenced on the DNBSEQ‐T7 platform (MGI Tech Co., Ltd., China). Raw sequencing reads were quality‐filtered using fastp (version 0.23.0). Cleaned reads were aligned to the Homo sapiens reference genome (GRCh38, NCBI) with subsequent quantification of exonic reads mapped to annotated genes using featureCounts (Subread‐1.5.1; Bioconductor).

The identification of differentially expressed genes was performed using the DESeq2 package.^[^ ^64^ ^]^ Subsequently, the ranked genes were submitted to the clusterProfiler package to conduct GSEA with reference gene sets from the molecular signature database (MSigDB).^[^ ^65^, ^66^, ^67^ ^]^ Gseavis package was used to visualize results. For GSVA, the TCGA‐BLCA dataset was submitted to the GSVA package to calculate the signaling score.^[^ ^68^ ^]^ The correlation between gene expression and score was visualized by the ggpubr package.

### Cell Proliferation Assay

Cells were pretreated and seeded at a density of 2000 cells per well in six replicate 96‐well plates. Following the addition of MTT solution, the plate would be placed in an incubator for 4 hrs. With removing culture supernatants, the wells were added 150 µL DMSO, following shaking fully, and detected by spectrophotometer (SpectraMax M2, USA) at 570 nm. The whole experiment lasted for the indicated time.

### Colony‐Formation Assays

A suspension of 1000 pretreated cells was seeded into a 6‐well plate and plated in incubator for 8–10 days. After removing supernatant culture, colonies in the well were handed with 4% paraformaldehyde fixative (BL539A, biosharp, China) for 1 hr, stained by 0.1% crystal violet solution for 1 hr, and captured by camera. Finally, the colonies were counted by the ImageJ software.

### Transwell Assay

Cells were pretreated as indicated. ≈600 µL medium was added to the 24‐well plates, and transwell chambers were put into wells. After suspended in 300 µL serum‐free medium, a total of 1 × 10^5^ 5637 cells and 4 × 10^4^ UM‐UC‐3 cells were seeded in the upper transwell chamber, following placing in the incubator at 37 °C for 24 hr. Then, removing all liquid, cells in the chamber were fixed in 4% PFA, followed by staining in 0.1% crystal violet solution for 1 hr. Next, the chambers were cleaned and dried out. Finally, migrated cells were captured by the microscope and subsequently quantified using ImageJ software.

### Wound Healing Assay

Cells were treated as indicated in 6‐well plates. When cells in the well were in confluent status, a sterile pipette tip was utilized to create a scratch on the cell monolayer. After washing by PBS gently, cells were captured by phase contrast microscopy, which would be set as 0 h. Subsequently, cells were cultured in medium without FBS for 24 h and finally captured. The calculation of the gap closure rate depended on the migration area through the ImageJ software.

### Cell Cycle Analysis

Pretreated BLCA cells as indicated, were harvested, followed by washing twice with PBS. The cell pellets were processed with a propidium iodide staining solution and permeabilization reagents according to the cell cycle staining kit (CCS012, Multi Sciences). After incubation, cell cycle distribution was evaluated using a Beckman Cytoflex flow cytometer, and data were processed via FlowJo software.

### Immunofluorescence Staining Analysis

Cells were seeded on slides in a 24‐well plate and treated as indicated. After washing with PBS once time, cells were fixed with 4% paraformaldehyde for 40 mins and treated with 0.3% Triton X‐100 and 2% bovine serum albumin solution for 1 hr. With three PBS washes, cells were incubated with primary antibody, secondary antibody, and DAPI, respectively. After staining, slides were rinsed briefly in PBS to remove residual reagents. Then, slides were mounted using an antifade mounting medium containing DAPI (DAPI Fluoromount‐G, 36308ES20, YEASEN, China) to preserve fluorescence signals and counterstain nuclei. Finally, mounted slides were stored away from light at 4 °C for 24 hrs to cure the medium and captured by the confocal laser microscope (C2+, Nikon, Japan).

Primary and secondary antibodies for immunofluorescence (IF) analysis used in this study were provided in Table S3 (Supporting Information).

### Lentivirus Infection

The lentiviruses packaged with shYKT6, shUSP7, and shNC were acquired from GenePharma (Shanghai, China), and sequences were provided in Table S4 (Supporting Information). BLCA cell lines were transfected with the mentioned shRNA in the presence of polybrene, following screened by 1 µg mL^−1^ puromycin (Sigma, USA).

### Animal Studies

Animal experiments in this study got ethical approval from the Experimental Animal Welfare and Ethics Committee of Zhongnan Hospital (approval number: ZN2023047). Male BALB/C‐nude mice (4 weeks old) were purchased from BIONT biological technology (Wuhan, China) and acclimated for one week. To establish a xenograft model, mice were distributed into two groups (n = 6) randomly, and ≈5 × 10^6^ shNC‐transfected and shYKT6‐transfected UM‐UC‐3 cells were implanted subcutaneously into mice. Since it comes to formation, the tumor was examined at three‐day intervals until the experiment ended. Following 28 days, the tumors were collected via surgery and weighed. The tumor volume was assessed using the formula Volume = 1/2 × length × width.^2^ In addition, tumor tissues were processed by H&E and immunohistochemistry (IHC). Furthermore, nude mice were distributed into two groups (n = 3) randomly to assess BLCA cell metastasis regulated by YKT6. ≈1 × 10^6^ shNC‐transfected and shYKT6‐transfected UM‐UC‐3 cells were injected via the tail vein into mice. Fluorescence intensity of lung metastasis was evaluated six weeks post‐injection via the IVIS Spectrum living imaging system. For the xenograft model of YKT6 overexpression, UM‐UC‐3 were transfected with lentiviruses containing YKT6 sequences and the vector (LV6). Subsequently, shNC and shUSP7 UM‐UC‐3 cells were established based on the construction of overexpressing YKT6 cells. ≈5 × 10^6^ LV6‐transfected and YKT6‐transfected UM‐UC‐3 cells were implanted subcutaneously into mice. Subsequently, mice with palpable tumors were treated intraperitoneally (i.p.) with P5091 (15 mg kg^−1^) twice a week with DMSO as controls. The subsequent tumor measurements were performed as described above.

### H&E Staining and Immunohistochemistry (IHC) Assay

Paraffin‐embedded xenograft tissue sections (5 µm) were processed for H&E staining. After dewaxing in xylene and rehydration through a graded ethanol series (100% to 70%) and water, sections were stained with 10% hematoxylin (Sigma–Aldrich, USA), rinsed, and counterstained with 1% eosin solution containing 0.2% glacial acetic acid. Sections were then dehydrated through a reverse ethanol gradient (70% to 100%) and cleared in xylene.

For IHC, paraffin‐embedded tissues were sectioned, deparaffinized, and rehydrated. Epitope retrieval was performed using citrate buffer (pH 6.0), and 0.3% H_2_O_2_ was utilized to inhibit endogenous peroxidase. Sections were sequentially incubated with primary and secondary antibodies, and signals were visualized through peroxidase‐conjugated DAB substrate. Images were acquired by an automatic upright microscope (Leica, Germany).

Primary and secondary antibodies for immunohistochemistry (IHC) analysis used in this study were provided in Table S3 (Supporting Information).

### Statistical Analysis

Statistical comparison between the two groups was assessed via a two‐tailed Student's t‐test or a Wilcoxon test. For multiple group comparison, differences were assessed by one‐way ANOVA followed by Sidak's or Dunnett's multiple comparisons test, depending on the specific situation. Kaplan‐Meier curves were generated to plot survival distributions, and the difference between groups was compared using the log‐rank test. Statistical significance was defined as *p* < 0.05 between groups, and the *p*‐value was shown for each statistical comparison result. Details of statistics and sample sizes were described in the figure legends. The statistical analyses were performed using the R software and GraphPad Prism.

### Declarations

### Declarations–Ethics Statement

For human samples, this study was performed in accordance with the Declaration of Helsinki and was approved by the Medical Ethics Committee of Zhongnan Hospital of Wuhan University (approval number: 2021125). The animal study was approved by the Experimental Animal Welfare Ethics Committee at Zhongnan Hospital of Wuhan University (approval number: ZN2023047).

## Conflict of Interest

The authors declare that they have no competing interests.

## Author Contributions

S.T., W.D., and Y.L. contributed equally to this work. Y.X., X.W., and K.Q. Designed and supervised the study. S.T., W.D., Y.L., J.S., T.L., K.X., F.Z., M.L., J.Y., G.W., and L.J. performed the most experiments. S.T., W.D., J.S., T.L., and S.C. performed the formal analysis. S.T., W.D., Y.L., J.S., T.L., M.J., Y.Z., and K.Q. Helped with data collection and assembly. S.T., L.J., and W.D. wrote the first draft. L.J., Y.X., X.W., and K.Q. Critically revised drafts of the manuscript. All authors reviewed the manuscript.

We uploaded the RNA‐seq data generated in this study to the Gene Expression Omnibus (GEO) database under accession number GSE293406. The GSE13507, GSE121711, and GSE31684 dataset was obtained from the GEO database (https://www.ncbi.nlm.nih.gov/geo/). The remaining data can be accessed in the article or in the Supplementary Information. Original blot images and other source data are provided with this paper.

## Supporting information



Supporting Information

Supporting Information

## Data Availability

The data that support the findings of this study are openly available in [Gene Expression Omnibus (GEO)] at [https://www.ncbi.nlm.nih.gov/geo/query/acc.cgi?acc = GSE293406], reference number [293406].
