# Supplementary material for: YKT6 Promotes Bladder Cancer Progression by Stabilizing β‐catenin Through USP7‐Mediated Deubiquitination
Source: Adv Sci (Weinh). 2025 Nov 26;13(8):e07166. doi: 10.1002/advs.202507166 (PMC12884800; doi:10.1002/advs.202507166)
Supplement: Supplementary file 1 — Supporting Information [file ADVS-13-e07166-s002.pdf]

Supplementary Information

YKT6 Promotes Bladder Cancer Progression by Stabilizing β-catenin through USP7-Mediated Deubiquitination

Figures S1-S9: Pages 2-17

Tables S1-S4: Pages 18-22

Figures S1-S9

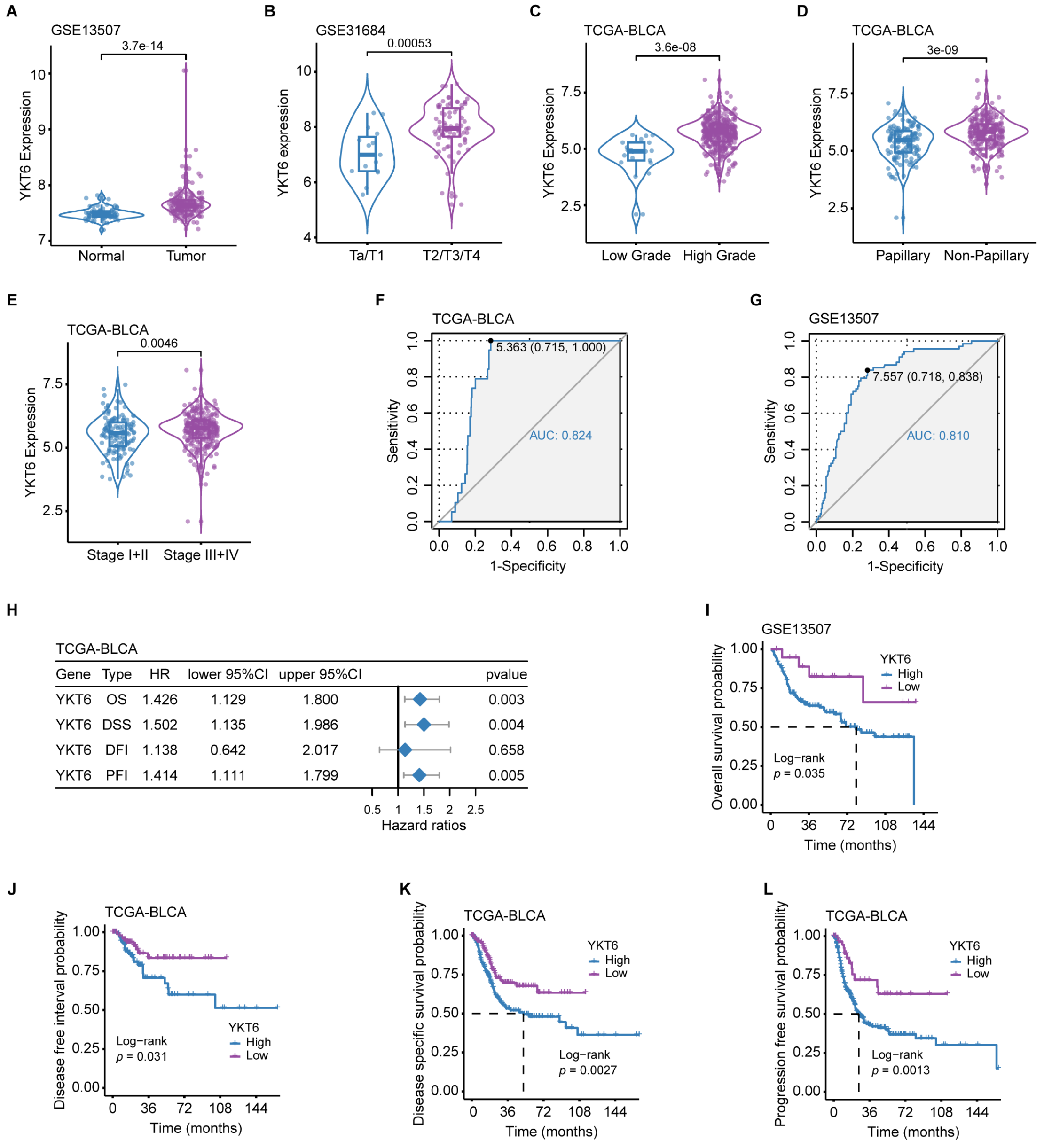

Figure S1. Upregulated YKT6 predicts poor prognosis in BLCA.

(A) Relative YKT6 expression in normal (n = 68) and tumor (n = 188) from GSE13507 cohort**. (B)** Relative YKT6 expression in different T stage from GSE31684 cohort (Ta/T1: n = 15; T2/T3/T4: n= 78). **(C-E)** YKT6 expression in different grade (C, low: n = 21; high: n = 379), subtype (D, papillary: n = 132; non-papillary: n = 266) and stage (E, stage I+II: n = 131; stage III+IV: n = 270) of BLCA in TCGA-BLCA. **(F-G)** ROC curve analysis of normal versus tumor based on YKT6 expression from TCGA database (F) and GSE13507 cohort (G). **(H)** Forest plots of univariable Cox regression analysis

for overall survival (OS), disease-specific survival (DSS), disease-free interval (DFI), and progression-free interval (PFI) based on YKT6 expression in TCGA-BLCA. **(I)** Kaplan-Meier survival curves showing reduced overall survival in BLCA patients with high YKT6 expression in GSE13507 cohort (n = 165). **(J-L)** Kaplan-Meier survival curves demonstrating reduced disease-free interval (J, n = 186), disease-specific survival (K, n = 388), and progression-free survival (L, n = 403) in BLCA patients with high YKT6 mRNA levels in TCGA-BLCA. Data are presented as mean values ± SD; Wilcoxon test (A-E); log-rank test for survival analysis (I-L).

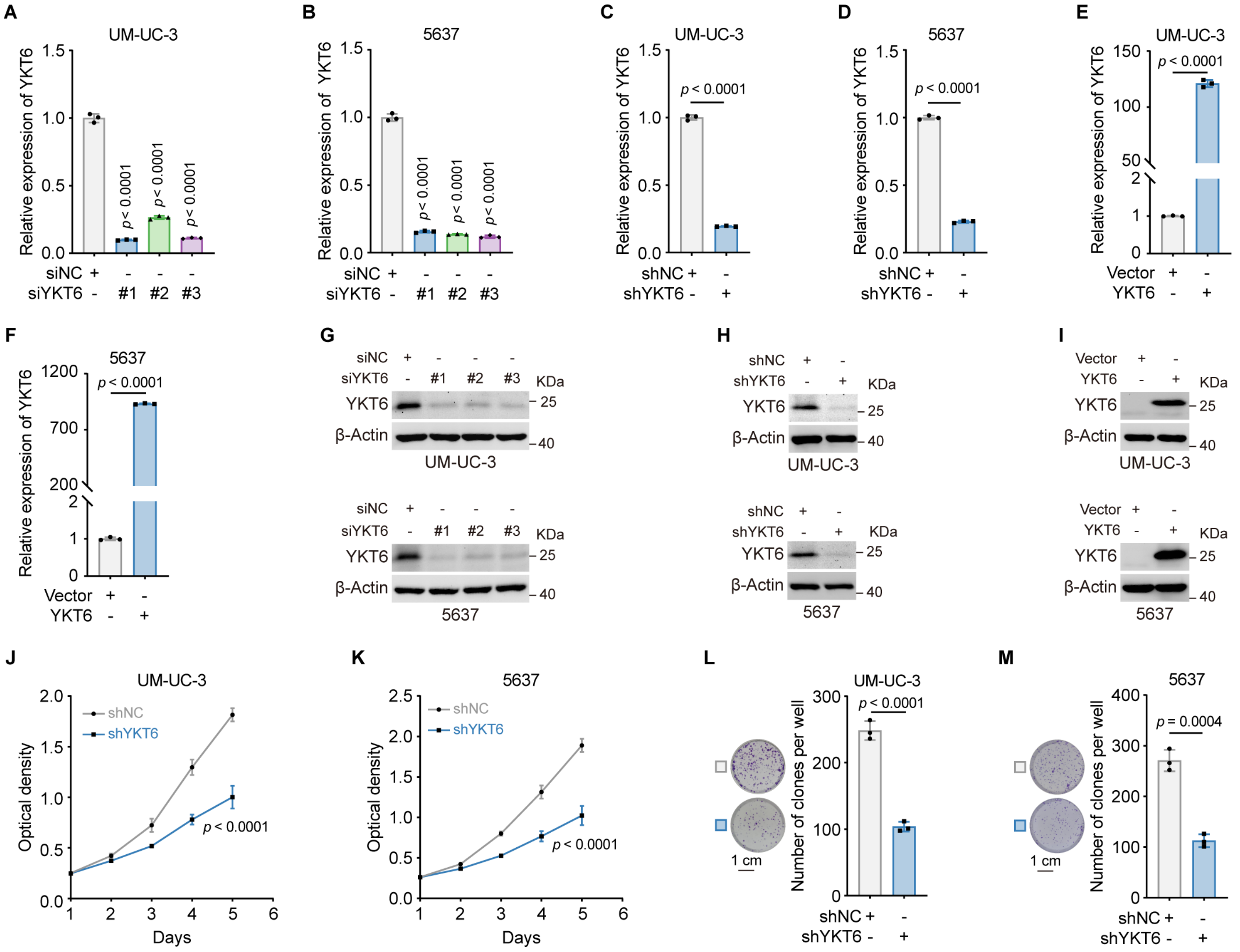
A
UM-UC-3
Relative expression of YKT6
siNC
siYKT6
p < 0.0001
B
5637
C
shNC
shYKT6
D
E
Vector
YKT6
F
G
KDa
β-Actin
H
I
J
Optical density
Days
K
L
Number of clones per well
1 cm
M
p = 0.0004

Figure S2. YKT6 deficiency inhibited BLCA cells proliferation.

(A-B) Knockdown efficiency of YKT6-targeting siRNAs in UM-UC-3 (A, n = 3 per group) and 5637 (B, n = 3 per group) cells. **(C-D)** Stable knockdown YKT6 efficiency using shRNA detected by qRT-PCR in UM-UC-3 (C, n = 3 per group) and 5637 (D, n = 3 per group) cells. **(E-F)** YKT6 overexpression efficiency in UM-UC-3 (E, n = 3 per group) and 5637 (F, n = 3 per group) cells. **(G-H)** Immunoblotting confirms reduced YKT6 protein levels in transient (G) and stable (H) knockdown in UM-UC-3 and 5637 cells. **(I)** Immunoblot validation of YKT6 overexpression in UM-UC-3 and 5637 cells. **(J-K)** Proliferation curves of shNC and shYKT6 cells assessed by MTT assay in UM-UC-3 (J, n = 6 per group) and 5637 (K, n = 6 per group) cells. **(L-M)** Representative image and statistic graph of YKT6 knockdown in UM-UC-3 (L, n = 3 per group) and 5637 (M, n = 3 per group) evaluated by colony formation assay. Scale bar: 1 cm. Data are presented as mean values ± SD; one-way ANOVA with Dunnett’s multiple comparisons test (A-B); two-tailed unpaired Student’s t-test (C-F, L-M); two-tailed unpaired Student’s t-test at Day 5 (J-K).

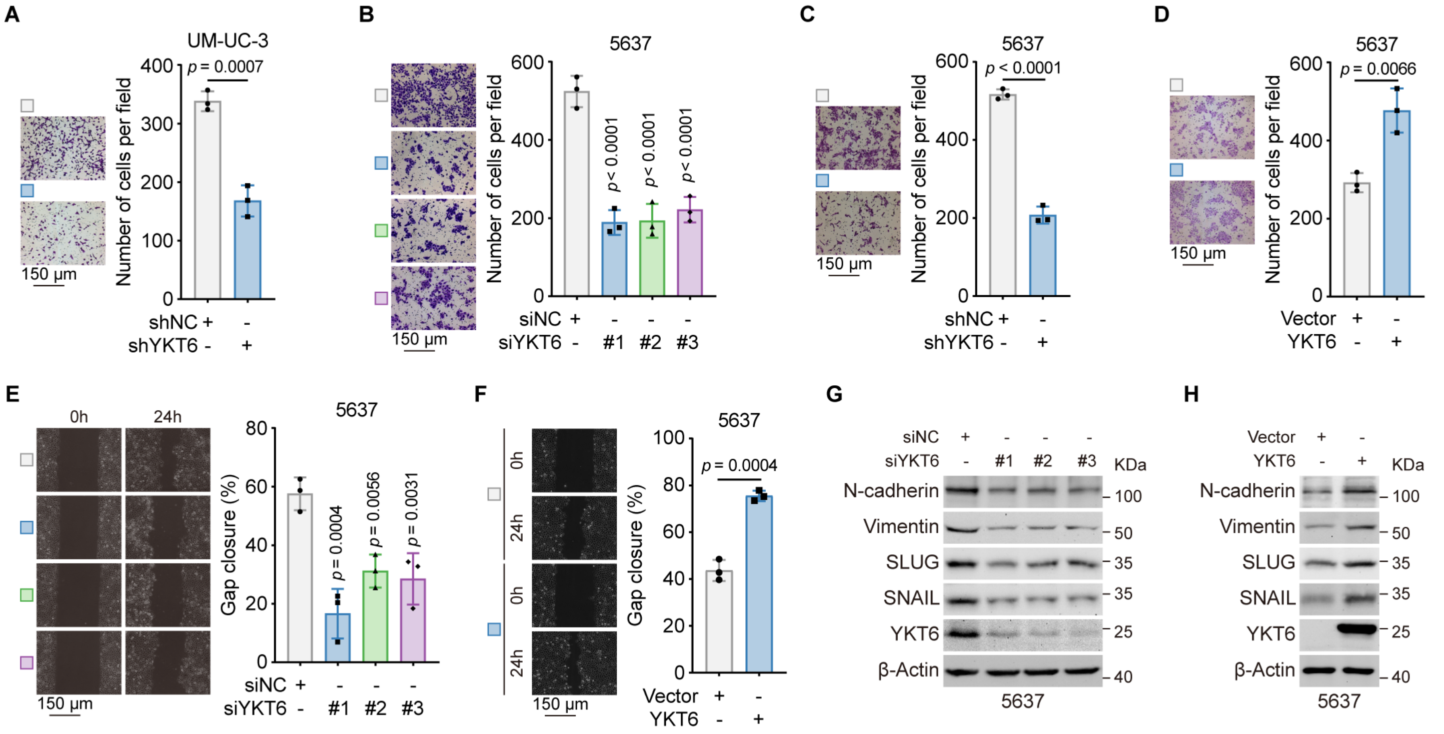

Figure S3. YKT6 inhibition suppressed BLCA cells migration.

(A) Representative image and statistic graph of shYKT6 transfected UM-UC-3 cells detected by transwell migration assay (n = 3 per group). Scale bar: 150 μm. **(B-D)** Representative images and statistic graphs after YKT6 knockdown or overexpression in 5637 cells (n = 3 per group). Scale bar: 150 μm. **(E-F)** The wound healing representative images and statistic graphs upon YKT6 knockdown or overexpression in 5637 cells (n = 3 per group). Scale bar: 150 μm. **(G-H)** The EMT-related protein levels of YKT6 knockdown or overexpression in 5637 cells lysate evaluated by immunoblot assay. Data are presented as mean values ± SD; one-way ANOVA with Dunnett's multiple comparisons test (B, E); two-tailed unpaired

Student’s t-test (A, C-D, F).

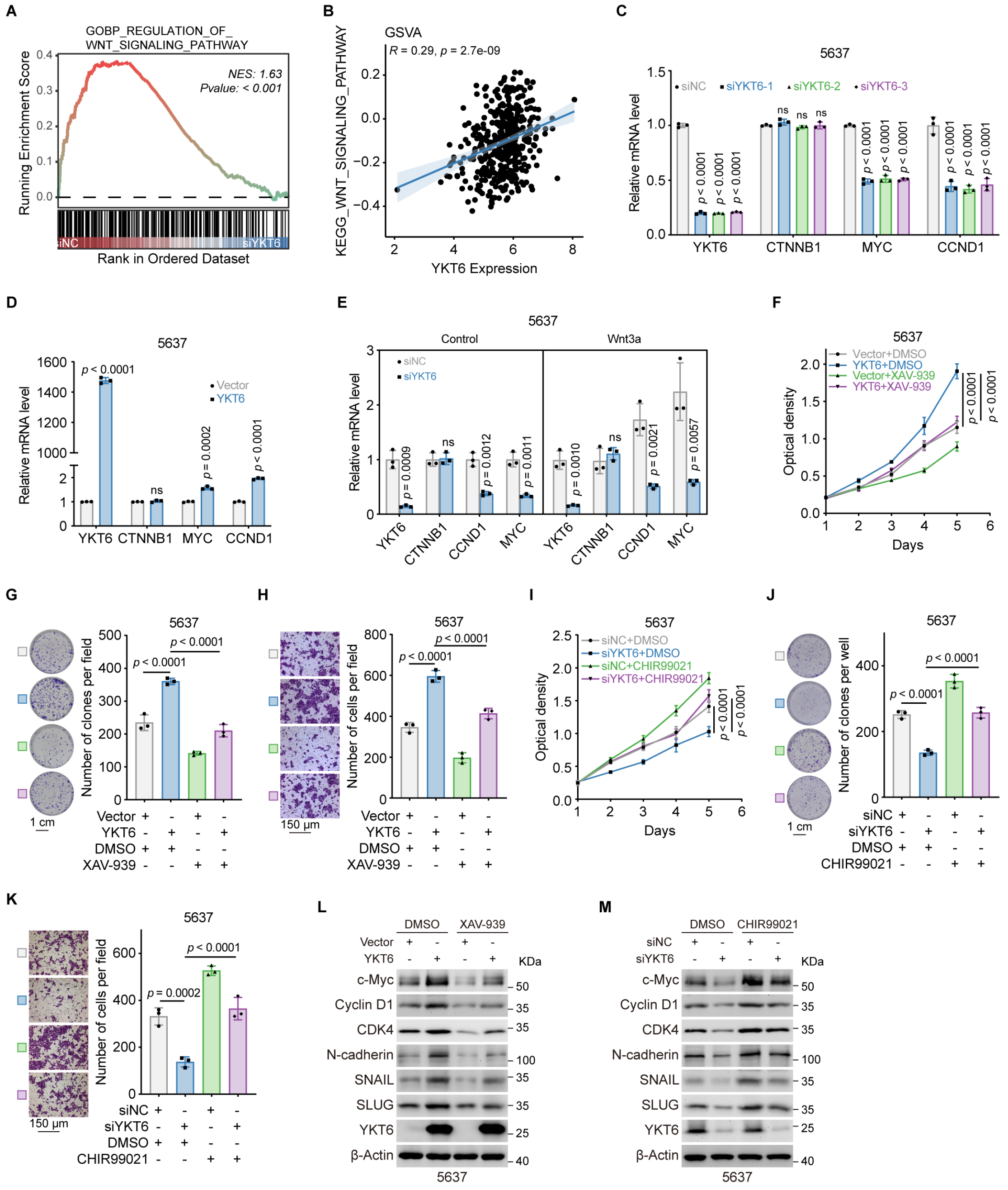

Figure S4. YKT6 modulates Wnt/β-catenin signaling activity in BLCA.

(A) GSEA of RNA-seq data from YKT6-silenced cells, showing enrichment in Wnt signaling regulation terms. **(B)** Positive correlation between YKT6 expression and KEGG Wnt pathway activation scores (GSVA) in the TCGA-BLCA datasets. **(C-D)** qRT-PCR analysis of β-catenin and target gene (*MYC*, *CCND1*) mRNA levels in 5637 cells following YKT6 knockdown (C, n = 3 per group) or overexpression (D, n = 3 per group). **(E)** Rescue of YKT6 knockdown-mediated suppression of Wnt targets by Wnt3a (100 ng/mL, n = 3 per group). **(F-H)** Pharmacological Wnt inhibition with XAV-939 (10 μM) reverses YKT6-driven proliferation (F, n = 6 per group), colony formation

(G, n = 3 per group, Scale bar: 1 cm), and migration (H, n = 3 per group, Scale bar: 150 μm) in 5637 cells. **(I-K)** Wnt activation via CHIR99021 (4 μM) restores proliferation (I, n = 6 per group), colony formation (J, n = 3 per group, Scale bar: 1 cm), and migration (K, n = 3 per group, Scale bar: 150 μm) in YKT6-silenced 5637 cells. **(L-M)** Immunoblot analysis confirms that XAV-939 (L) or CHIR99021 (M) counteracts YKT6-mediated changes in downstream effectors. Data are presented as mean values ± SD; two-tailed unpaired Student's t-test (D, E); one-way ANOVA with Dunnett's multiple comparisons test (C); one-way ANOVA with Sidak's multiple comparisons test at Day 5 (F, I); one-way ANOVA with Sidak's multiple comparisons test (G-H, J-K).

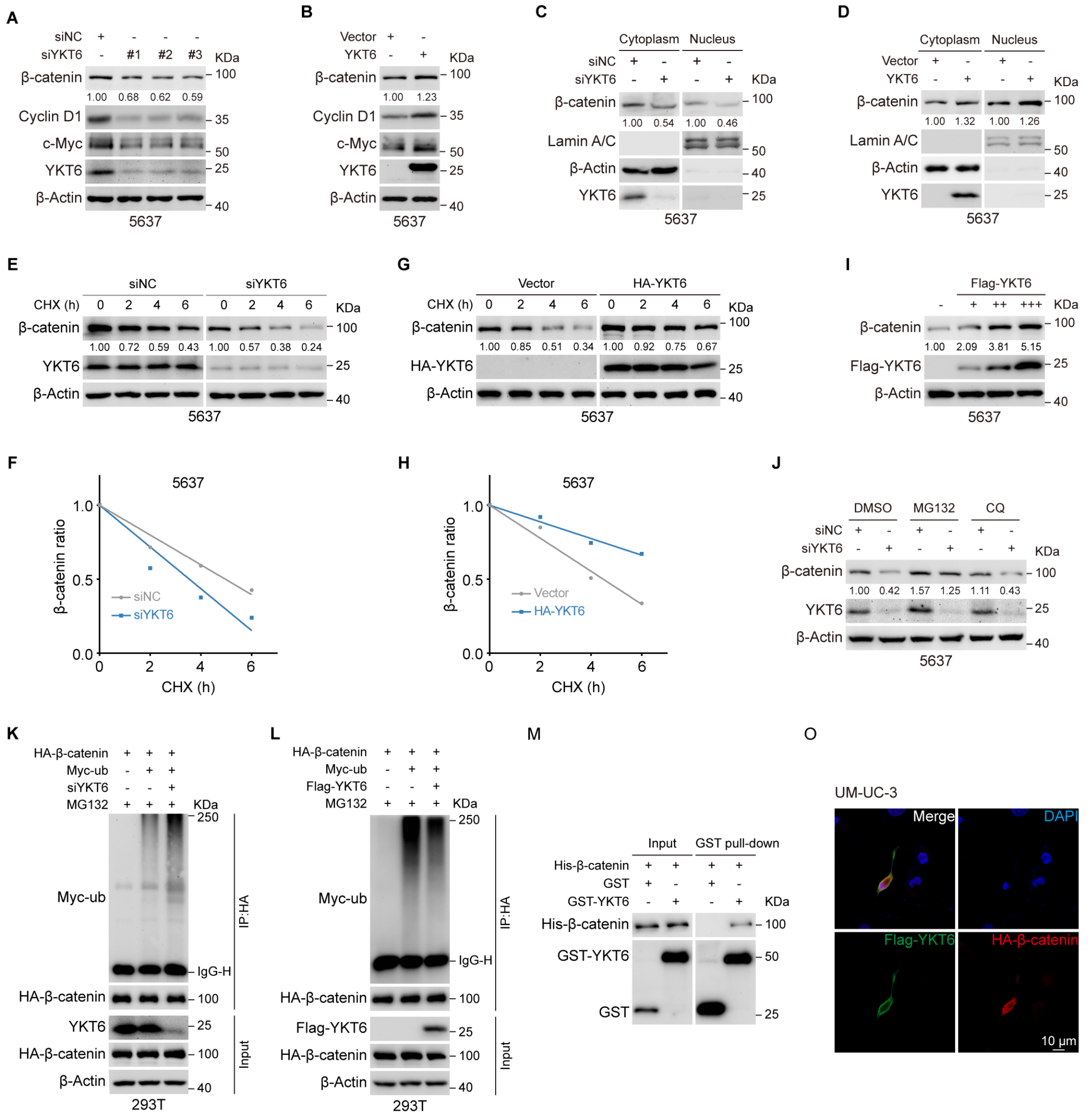

Figure S5. YKT6 enhances β-catenin stability.

(A-B) Immunoblot analysis of β-catenin and downstream targets protein level in 5637 cells following YKT6 knockdown (A) or overexpression (B). **(C-D)** Subcellular fractionation and Western blotting of cytoplasmic and nuclear β-catenin in 5637 cells with YKT6 knockdown (C) or overexpression (D). Lamin A/C and β-Actin served as nuclear and cytoplasmic markers, respectively. **(E-F)** YKT6 knockdown effects on the degradation of β-catenin protein upon cycloheximide (CHX, 50 μg/mL) treatment for indicated times in 5637 cells. **(G-H)** YKT6 overexpression effects on the degradation of β-catenin protein upon cycloheximide (CHX, 50 μg/mL) treatment for indicated times in 5637 cells. **(I)** Dose-dependent increase in β-catenin protein levels upon graded YKT6 overexpression. **(J)** The β-catenin protein changes after YKT6 knockdown with MG132 (10 μM) or CQ (100 μM) treatment in 5637 cells. **(K-L)** Ubiquitination of β-

catenin protein upon YKT6 knockdown (K) or overexpression (L) in 293T cells. **(M)** Immunoblot result of GST-YKT6 and His-β-catenin from GST pull-down assay. **(O)** Co-localization of Flag-YKT6 and HA-β-catenin in UM-UC-3 cell. Scale bar: 10 μm.

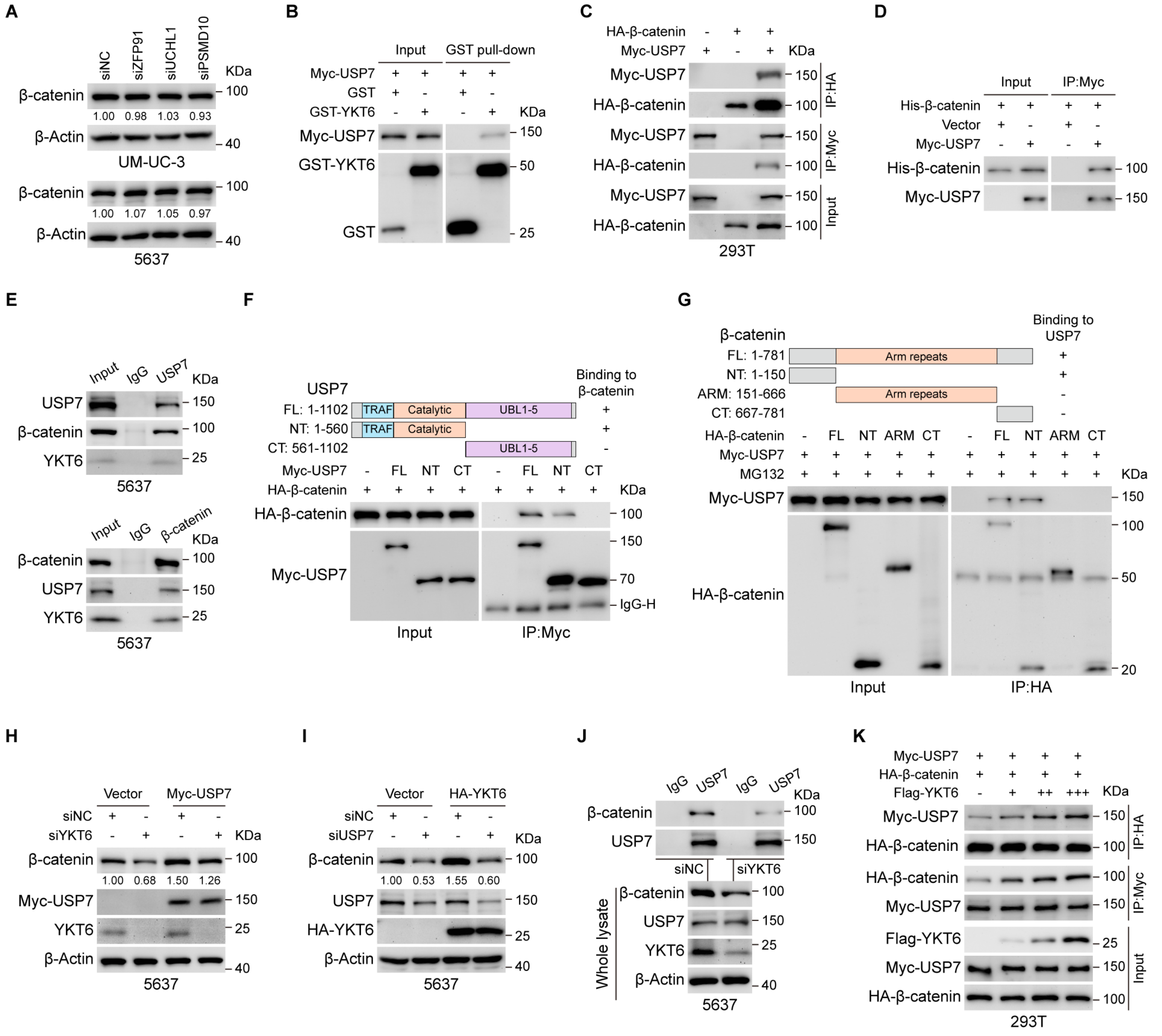
A
siNC
siZFP91
siUCHL1
siPSMD10
KDa
β-catenin
1.00 0.98 1.03 0.93
β-Actin
UM-UC-3
β-catenin
1.00 1.07 1.05 0.97
β-Actin
5637
B
Input
GST pull-down
Myc-USP7
GST
GST-YKT6
Myc-USP7
GST-YKT6
GST
KDa
150
50
25
C
HA-β-catenin
Myc-USP7
KDa
Myc-USP7
HA-β-catenin
IP:HA
IP:Myc
Input
293T
D
Input
IP:Myc
His-β-catenin
Vector
Myc-USP7
His-β-catenin
Myc-USP7
100
150
E
Input
IgG
USP7
KDa
USP7
β-catenin
YKT6
5637
Input
IgG
β-catenin
KDa
β-catenin
USP7
YKT6
5637
F
USP7
Binding to β-catenin
FL: 1-1102
NT: 1-560
CT: 561-1102
TRAF
Catalytic
UBL1-5
Myc-USP7
HA-β-catenin
FL NT CT
KDa
HA-β-catenin
Myc-USP7
IgG-H
Input
IP:Myc
G
β-catenin
Binding to USP7
FL: 1-781
NT: 1-150
ARM: 151-666
CT: 667-781
Arm repeats
HA-β-catenin
Myc-USP7
MG132
FL NT ARM CT
KDa
Myc-USP7
HA-β-catenin
Input
IP:HA
H
Vector
Myc-USP7
siNC
siYKT6
KDa
β-catenin
1.00 0.68 1.50 1.26
Myc-USP7
YKT6
β-Actin
5637
I
Vector
HA-YKT6
siNC
siUSP7
KDa
β-catenin
1.00 0.53 1.55 0.60
USP7
HA-YKT6
β-Actin
5637
J
IgG
USP7
KDa
β-catenin
USP7
siNC
siYKT6
Whole lysate
β-catenin
USP7
YKT6
β-Actin
5637
K
Myc-USP7
HA-β-catenin
Flag-YKT6
++
+++
KDa
Myc-USP7
HA-β-catenin
IP:HA
IP:Myc
Flag-YKT6
Input
293T

Figure S6. β-catenin stabilization mediated by YKT6 depends on USP7.

(A) Immunoblot analysis detected the β-catenin protein level based on knockdown of ZFP91, UCHL1, and PSMD10. **(B)** The interaction between GST-YKT6 and Myc-USP7 was detected by GST pull-down assay. **(C)** Co-IP assay confirms the interaction between USP7 and β-catenin in 293T cell. **(D)** Immunoblot analysis of the interaction between His-β-catenin and Myc-USP7. **(E)** Endogenous IP of USP7 and β-catenin antibody detecting the interaction among YKT6, USP7, and β-catenin in 5637 cells. **(F)** The interaction between HA-β-catenin and the full-length and truncations of Myc-USP7 detected by immunoblot assay. **(G)** The interaction between Myc-USP7 and the full-length and truncations of HA-β-catenin detected by immunoblot assay. **(H)** The β-catenin protein level after YKT6 knockdown with or without USP7 overexpression in 5637. **(I)** The β-catenin protein level after USP7 knockdown with or without YKT6 overexpression in 5637. **(J)** The interaction between USP7 and β-catenin upon YKT6 knockdown detected by endogenous co-IP assay in 5637. **(K)** The interaction between USP7 and β-catenin upon YKT6 gradient overexpression detected by exogenous co-IP assay in 293T.

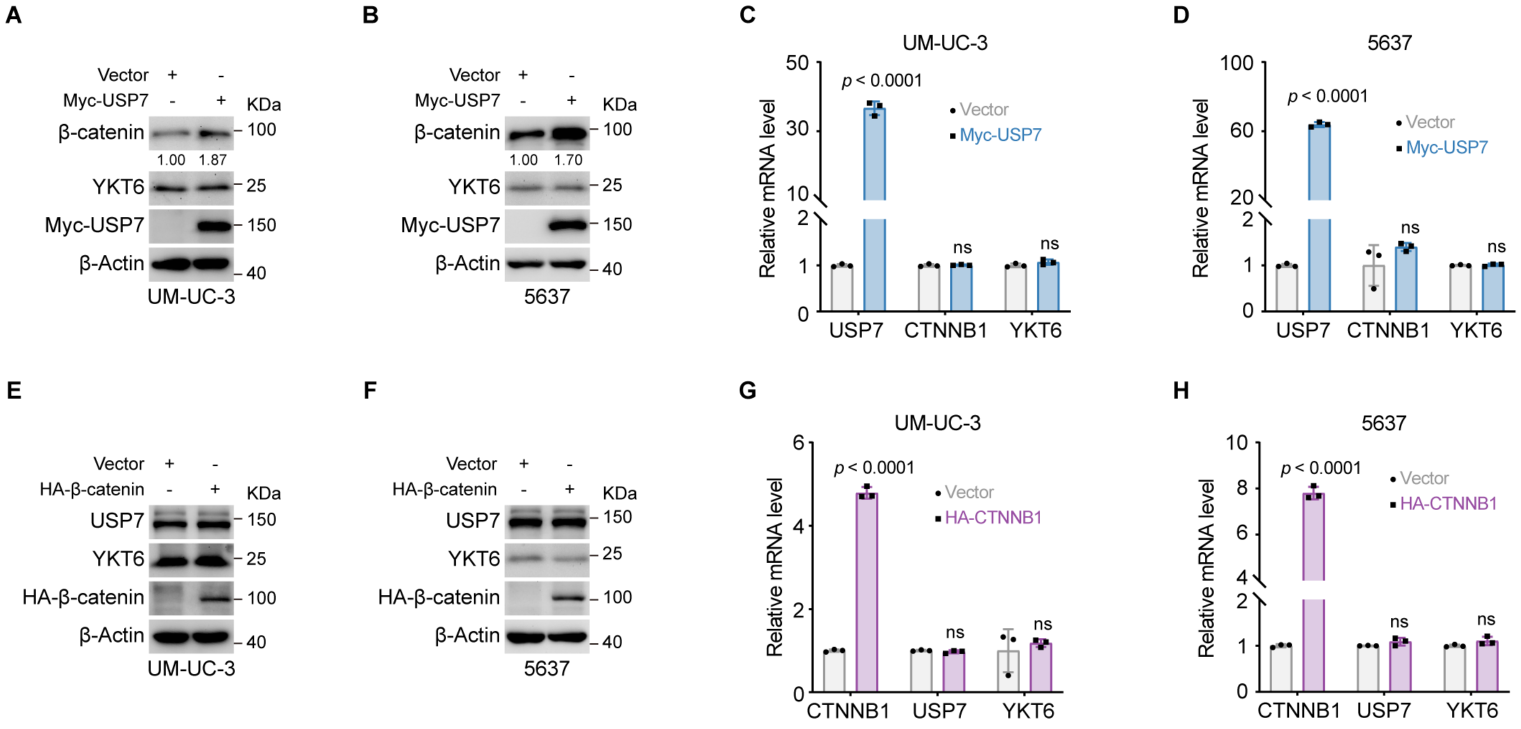

Figure S7. The relationship among YKT6, USP7 and β-catenin.

(A-B) Immunoblot analysis of YKT6 and β-catenin protein levels following USP7 overexpression in UM-UC-3 (A) and 5637 (B) cells. **(C-D)** qRT-PCR analysis reveals unchanged mRNA levels of YKT6 and β-catenin after USP7 overexpression in UM-UC-3 (C, n = 3 per group) and 5637 (D, n = 3 per group) cells. **(E-F)** Immunoblot analysis of YKT6 and USP7 protein levels in UM-UC-3 (E) and 5637 (F) cells overexpressing β-catenin. **(G-H)** qRT-PCR confirms unaltered YKT6 and USP7 mRNA levels in β-catenin-overexpressing UM-UC-3 (G, n = 3 per group) and 5637 (H, n = 3 per group) cells. Data are presented as mean values ± SD; two-tailed unpaired Student's t-test (C-D, G-H).

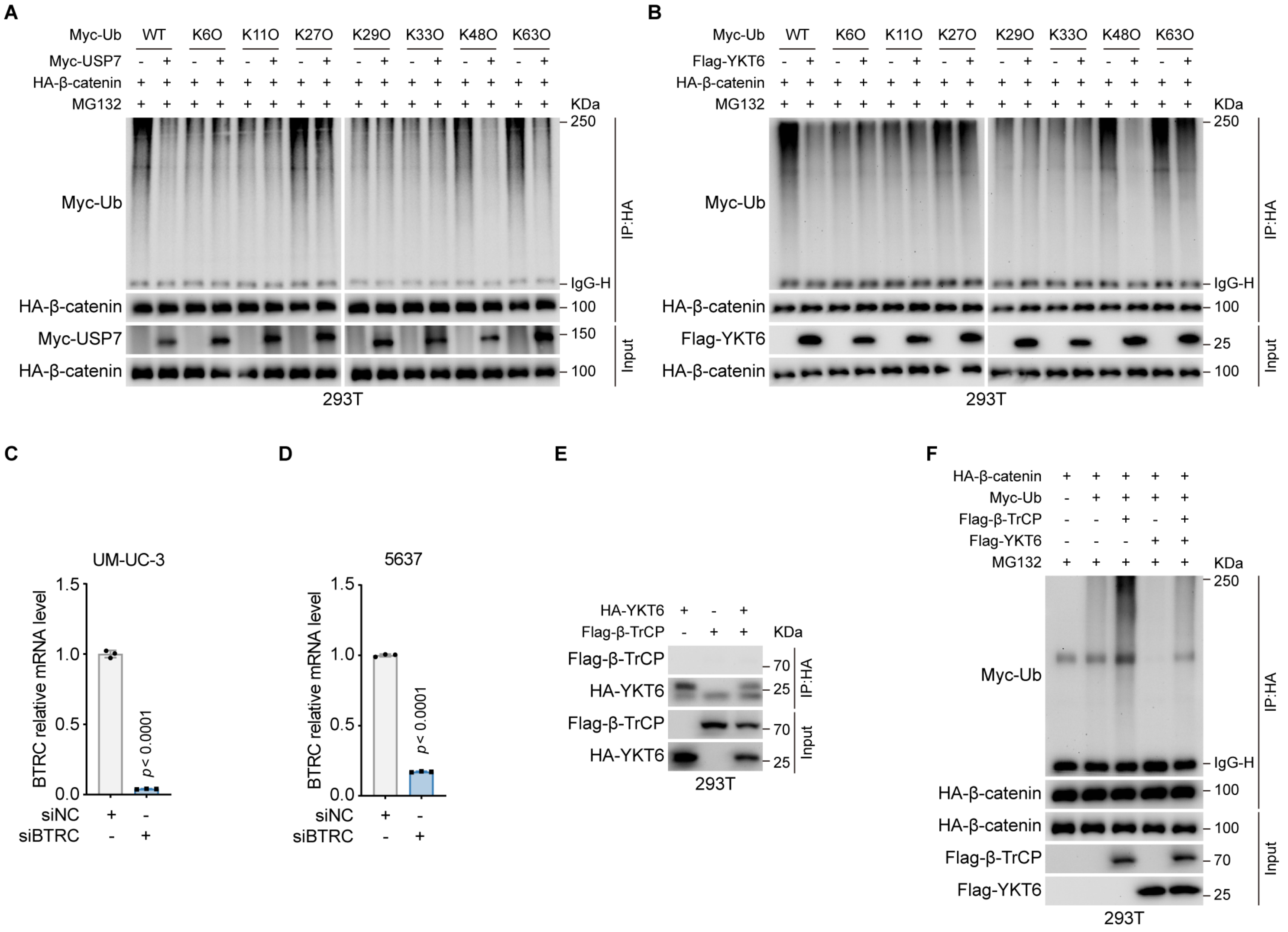
A
Myc-Ub
WT
K6O
K11O
K27O
K29O
K33O
K48O
K63O
Myc-USP7
HA-β-catenin
MG132
KDa
250
IgG-H
100
150
Myc-Ub
HA-β-catenin
Myc-USP7
IP:HA
Input
293T
B
Flag-YKT6
25
Flag-YKT6
C
UM-UC-3
BTRC relative mRNA level
1.5
1.0
0.5
0.0
p < 0.0001
siNC
siBTRC
D
5637
E
HA-YKT6
Flag-β-TrCP
70
F
Flag-YKT6

Figure S8. YKT6 promotes β-catenin deubiquitination via USP7.

(A) 293T cells were transfected with combinations of plasmids expressing Myc-USP7, HA-β-catenin and WT ubiquitin or the K6-, K11-, K27-, K29-, K33-, K48-, or K63-only ubiquitin mutant and were then treated with 10 μM MG132 for 8 hrs. **(B)** 293T cells were transfected indicated plasmids and treated with 10 μM MG132 for 8 hrs, following ubiquitin assay. **(C-D)** Validation of BTRC knockdown evaluated by qRT-PCR (n = 3 per group). **(E)** Co-IP analysis between HA-YKT6 and Flag-β-TrCP. **(F)** Ubiquitination of β-catenin upon overexpressing Flag-β-TrCP or Flag-YKT6 with 10 μM MG132 for 8 hrs. Data are presented as mean values ± SD; two-tailed unpaired Student's t-test (C-D).

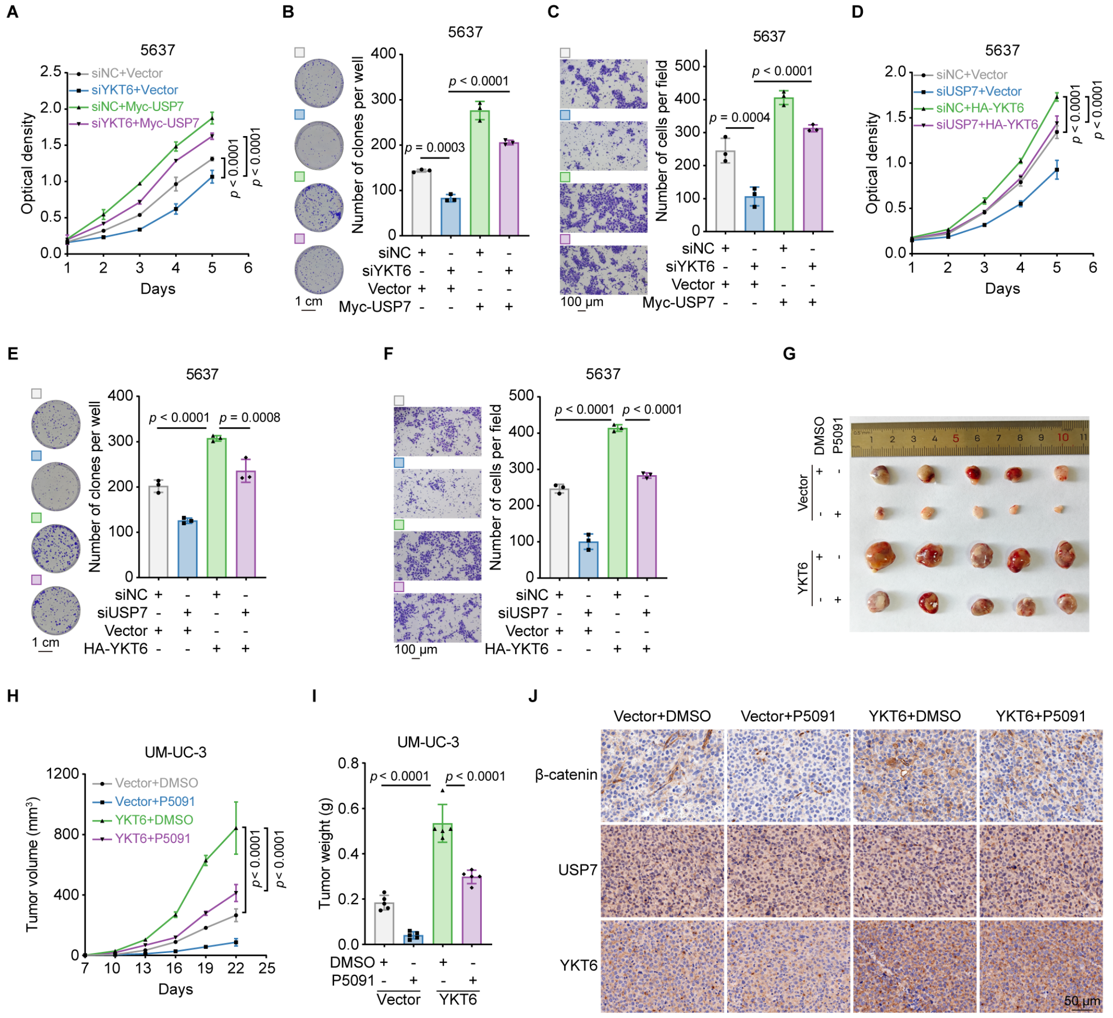

Figure S9. YKT6-USP7-β-catenin axis promotes BLCA progression.

(A-C) Overexpression of USP7 reverses YKT6-knockdown-driven proliferation (A, n = 6 per group), colony formation (B, n = 3 per group, Scale bar: 1 cm), and migration (C, n = 3 per group, Scale bar: 100 μm) in 5637 cells. **(D-F)** Knockdown of USP7 impairs YKT6-overexpression-driven proliferation (D, n = 6 per group), colony formation (E, n = 3 per group, Scale bar: 1 cm), and migration (F, n = 3 per group, Scale bar: 100 μm) in 5637 cells. **(G)** Image of subcutaneous tumors in xenograft models of YKT6-overexpressing UM-UC-3 cells with DMSO or P5091 (15 mg/kg) treatment. **(H-I)** The tumor growth curve (H) and weight (I) of indicated groups from xenograft model (n = 5 per group). **(J)** IHC staining of YKT6, USP7, and β-catenin from subcutaneous tumors in xenograft models (Scale bar: 50 μm). Data are presented as mean values ± SD; one-way ANOVA with Sidak's multiple comparisons test at Day 5 (A, D); one-way ANOVA with Sidak's multiple comparisons test (B-C, E-F, I); one-way ANOVA with Sidak's multiple comparisons test at Day 22 (H).

Tables S1-S4

Table S1. Clinicopathological characteristics of the ZNWU in-house dataset.

Patient	Gender	Age	Invasiveness	Grade	TNM stage	Neoadjuvant therapy	Survival status	Time
Patient-1	Male	69	MIBC	H	T4N0Mx	No	Alive	60
Patient-2	Male	68	MIBC	H	T2aN0Mx	No	Alive	60
Patient-3	Male	68	MIBC	H	T2bN1Mx	No	Dead	27
Patient-4	Male	79	NMIBC	L	T1N0Mx	No	Alive	58
Patient-5	Female	76	MIBC	H	T2bN0Mx	No	Dead	17
Patient-6	Male	67	MIBC	H	T4aN2Mx	No	Dead	10
Patient-7	Male	70	MIBC	H	T2N0Mx	No	Alive	55
Patient-8	Male	75	MIBC	H	T2bNxMx	Yes	Alive	55
Patient-9	Male	87	MIBC	H	T2N0Mx	No	Dead	31
Patient-10	Male	80	MIBC	H	T2N0MX	No	Dead	49
Patient-11	Male	69	NMIBC	L	T1N0Mx	No	Alive	50
Patient-12	Male	84	MIBC	H	T3aN0Mx	No	Dead	8
Patient-13	Male	65	MIBC	H	T2N0Mx	No	Alive	48
Patient-14	Female	63	NMIBC	H	T1N0Mx	No	Dead	37
Patient-15	Male	57	NMIBC	H	T1N0Mx	No	Alive	46
Patient-16	Male	71	MIBC	H	T3aN1Mx	No	LTFU	36
Patient-17	Male	64	NMIBC	H	T1NxMx	No	Alive	45

**Patient**	**Gender**	**Age**	**Invasiveness**	**Grade**	**TNM stage**	**Neoadjuvant therapy**	**Survival status**	**Time**
Patient-18	Male	65	NMIBC	H	TisN0Mx	No	Alive	44
Patient-19	Male	73	MIBC	H	T3N0Mx	No	Dead	25
Patient-20	Male	69	NMIBC	H	T1N0Mx	No	Alive	43
Patient-21	Male	74	MIBC	H	T3N0Mx	No	Alive	39
Patient-22	Female	47	NMIBC	H	T1N0Mx	No	LTFU	34
Patient-23	Male	73	MIBC	H	T2N0Mx	No	Alive	37
Patient-24	Male	50	NMIBC	H	T1N0Mx	No	LTFU	30
Patient-25	Male	82	NMIBC	H	T1N0Mx	No	Alive	34
Patient-26	Female	55	NMIBC	H	T1N0Mx	No	Dead	14
Patient-27	Male	78	MIBC	H	T4N1Mx	No	Dead	1
Patient-28	Female	67	NMIBC	L	T1N0Mx	No	Alive	47
Patient-29	Male	93	MIBC	H	T4aNxMx	No	Dead	8
Patient-30	Male	84	MIBC	H	T3aN0Mx	No	Alive	33
Patient-31	Male	75	MIBC	H	T2N1Mx	No	Alive	34
Patient-32	Male	59	MIBC	H	T2aN0Mx	No	Alive	34
Patient-33	Male	70	NMIBC	H	T1N0Mx	No	LTFU	20
Patient-34	Female	82	MIBC	H	T3N2Mx	No	Dead	14
Patient-35	Male	55	NMIBC	H	T1N0Mx	No	Alive	36
Patient-36	Male	72	MIBC	H	T4aN0Mx	No	Dead	6

LTFU: Lost to follow-up.

Table S2. Primer sequences for qRT-PCR.

Gene	Forward primer (5'-3')	Reverse primer (5'-3')
ACTB	CATGTACGTTGCTATCCAGGC	CTCCTTAATGTCACGCACGAT
YKT6	CAGCGTCCTCTACAAAGGCG	ACAATCAGTTGACTCGTGAAGG
CTNNB1	CATCTACACAGTTTGATGCTGCT	GCAGTTTTGTCAGTTCAGGGA
CCND1	GCTGCGAAGTGGAAACCATC	CCTCCTTCTGCACACATTTGAA
MYC	GGCTCCTGGCAAAAGGTCA	CTGCGTAGTTGTGCTGATGT
USP7	GGAAGCGGGAGATACAGATGA	AAGGACCGACTCACTCAGTCT
ZFP91	TGAGACCTACAAACCCCACTT	CCTTTTGGGTAAACGTGGACTTT
UCHL1	CCTGTGGCACAATCGGACTTA	CATCTACCCGACATTGGCCTT
PSMD10	GGGTGTGTGTCTAACCTAATGG	GGCCAGAATACTCTCCTTCAACT
BTRC	CCAGACTCTGCTTAAACCAAGAA	GGGCACAATCATACTGGAAGTG

Table S3. Details of antibodies used in this study.

Antibody	Catalog No.	Source	Dilution or amount	RRID
Flag-tag	F1804	Sigma	IP/1 μg; WB/1:1000	AB_262044
Flag-tag	20543-1-AP	Proteintech	IF/1:200	AB_11232216
HA-tag	TA180128	Origene	IP/1 μg; WB/1:1000; IF/1:200	AB_2622290
Myc-tag	AE010	ABclonal	IP/1 μg; WB/1:1000; IF/1:100	AB_2770408
β-Actin	66009-1-Ig	Proteintech	WB/1:20000	AB_2687938
β-Actin	sc-47778	Santa Cruz	WB/1:1000	AB_626632
N-Cadherin	13116	CST	WB/1:1000	AB_2687616
Vimentin	5741	CST	WB/1:1000	AB_10695459
SLUG	9585	CST	WB/1:1000	AB_2239535
SNAIL	3879	CST	WB/1:1000	AB_2255011
β-catenin	ab32572	Abcam	WB/1:1000; IHC/1:200	AB_725966
Cyclin D1	ab134175	Abcam	WB/1:1000	AB_2750906
c-Myc	ab32072	Abcam	WB/1:1000	AB_731658
CDK4	ab108357	Abcam	WB/1:1000	AB_10867218
CDK6	13331	CST	WB/1:1000	AB_2721897
YKT6	PA5-56565	Invitrogen	WB/1:500; IHC/1:50	AB_2649728
USP7	66514-1-Ig	Proteintech	WB/1:5000; IHC/1:500	AB_2881877
Ki-67	ab16667	Abcam	IHC/1:200	AB_302459

RRID: Research Resource Identifier;
WB: Western blot;
IP: Immunoprecipitation;
IHC: Immunohistochemistry;
IF: Immunofluorescence.

Table S4. The sequences of siRNA and shRNA.

siRNA	Sequence (5'-3')
siNC	UUCUCCGAACGUGUCACGUTT
siYKT-1	GCCUCAGCGUCCUCUACAAAG
siYKT-2	GGAGAAGGUACUAGAUGAAUU
siYKT-3	CAAGCAAGUCGACAGGAUAGA
siUSP7-1	ACCCUUGGACAAUAUUCCUTT
siUSP7-2	CCUGGAUUUGUGGUUACGUUA
siBTRC	AAGUGGAAUUUGUGGAACAUC
siZFP91	GAACUCAGAUAUACUCGGUTT
siUCHL1	AAGUUAGUCCUAAAGUGUATT
siPSMD10	GGUUGGUCUCCUCUUCAUATT

shRNA	Sequence (5'-3')
shNC	TTCTCCGAACGTGTCACGTTT
shYKT6	GCCTCAGCGTCCTCTACAAAG
shUSP7	CCTGGATTTGTGGTTACGTTA
