# Supplementary material for: YKT6 Promotes Bladder Cancer Progression by Stabilizing β‐catenin Through USP7‐Mediated Deubiquitination
Source: Adv Sci (Weinh). 2025 Nov 26;13(8):e07166. doi: 10.1002/advs.202507166 (PMC12884800; doi:10.1002/advs.202507166)
Supplement: Supplementary file 2 — Supporting Information [file ADVS-13-e07166-s001.zip › advs73011-sup-0002-data/Link to raw RNA sequencing data.pdf]

Information for reviewers

To access our RNA sequencing data sets uploaded in the Gene Expression Omnibus (GEO) database under the accession number GSE293406:

Reviewer access:

Go to https://www.ncbi.nlm.nih.gov/geo/query/acc.cgi?acc=GSE293406

Enter token **ctuzsycqlhkhpwz** into the box.
