# Supplementary material for: YKT6 Promotes Bladder Cancer Progression by Stabilizing β‐catenin Through USP7‐Mediated Deubiquitination
Source: Adv Sci (Weinh). 2025 Nov 26;13(8):e07166. doi: 10.1002/advs.202507166 (PMC12884800; doi:10.1002/advs.202507166)

Fig. 3J

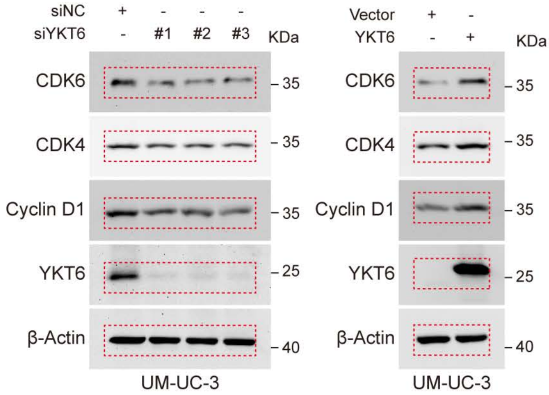

Fig. 3K

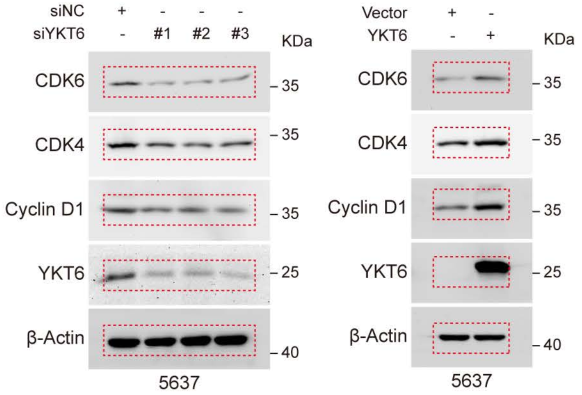

Fig. 4J

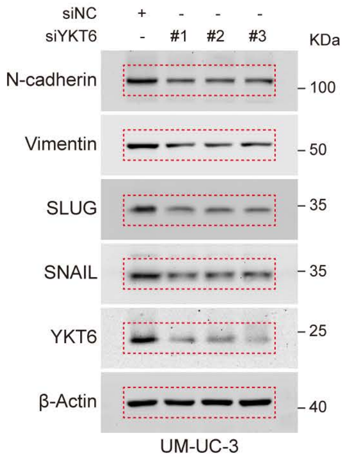

Fig. 4K

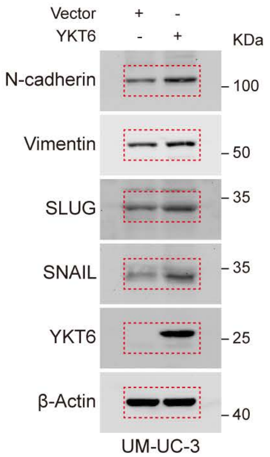

Fig. 5L

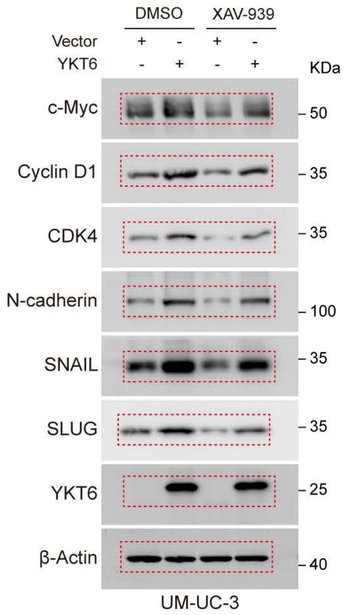

Fig. 5M

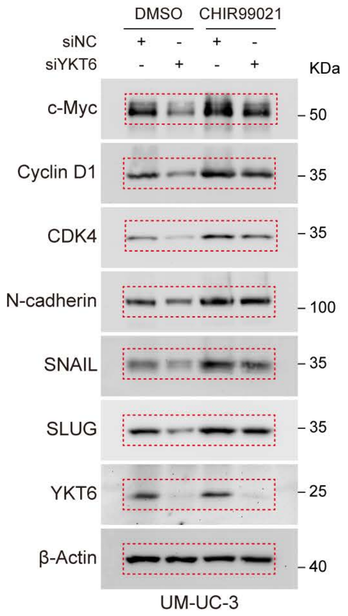

Fig. 6A

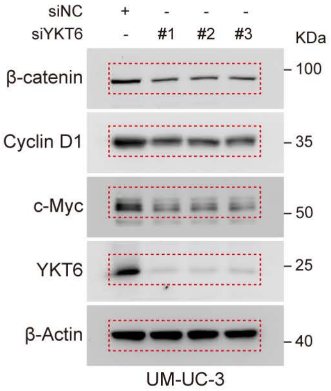

Fig. 6B

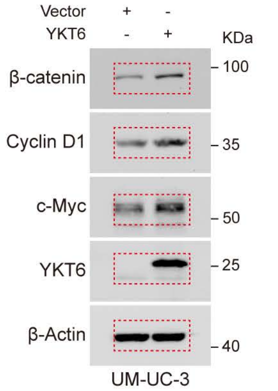

Fig. 6C

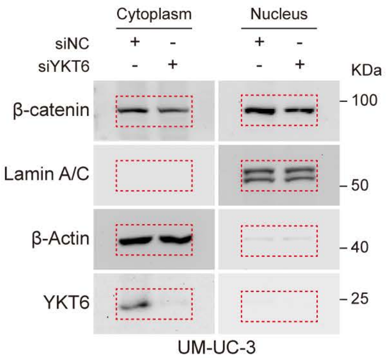

Fig. 6D

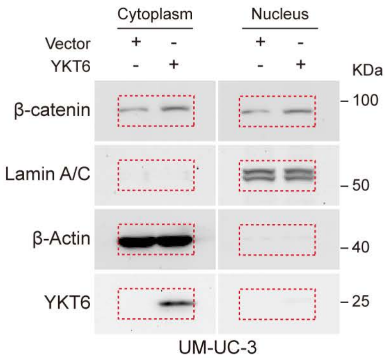

Fig. 6E

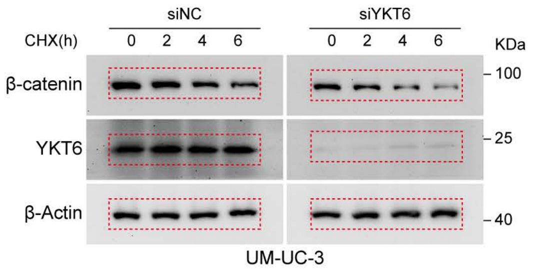

Fig. 6G

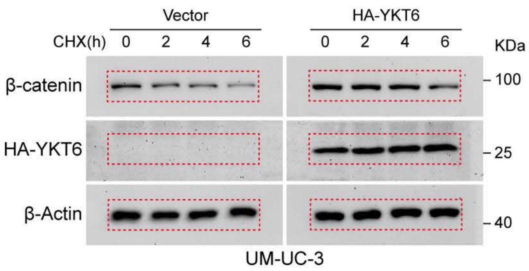

Fig. 6I

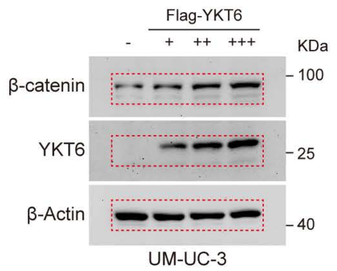

Fig. 6J

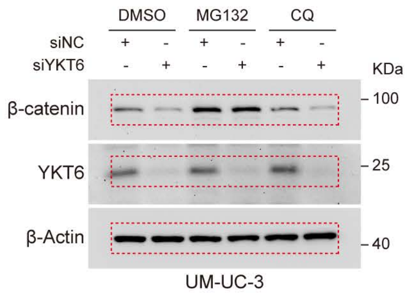

Fig. 6K

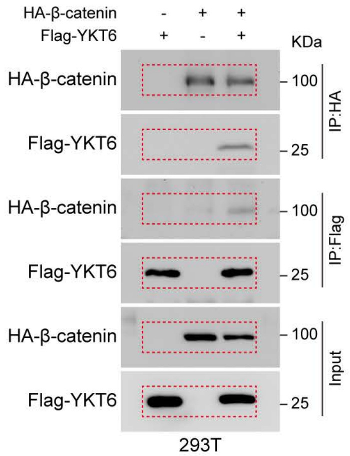

Fig. 6L

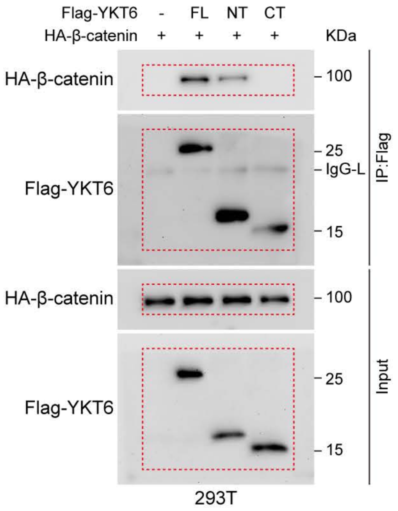

Fig. 6M

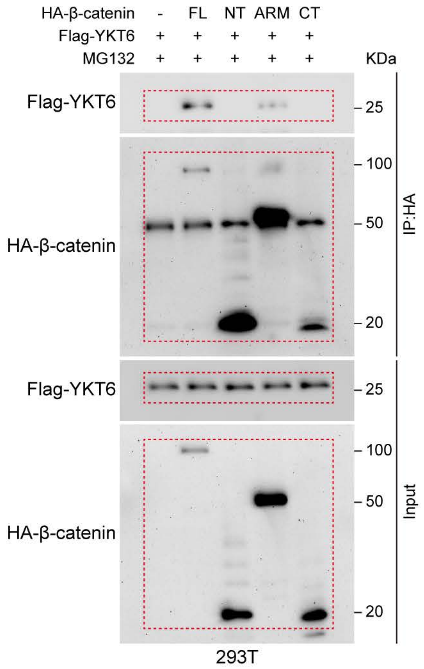

Fig. 7B

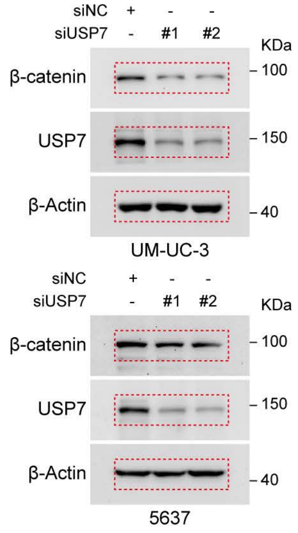

Fig. 7C

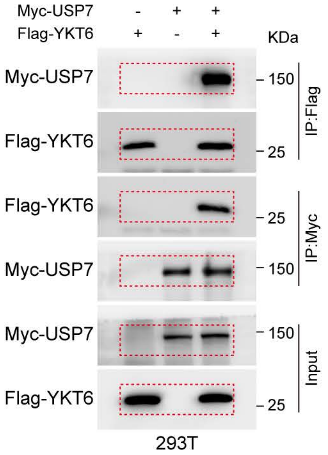

Fig. 7E

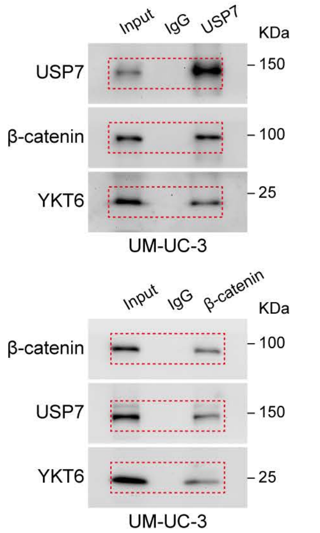

Fig. 7F

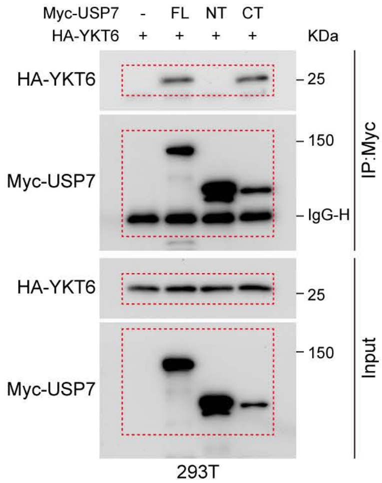

Fig. 7G

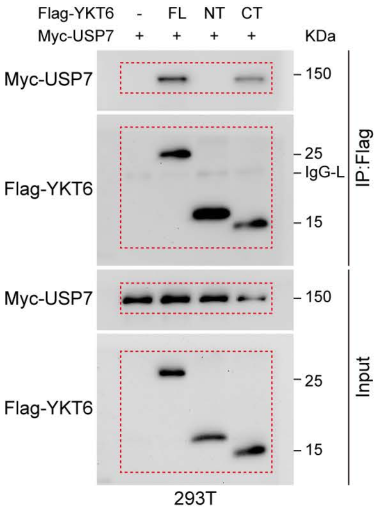

Fig. 7H

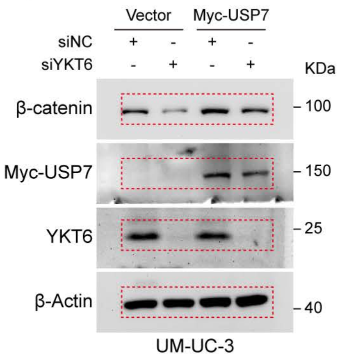

Fig. 7I

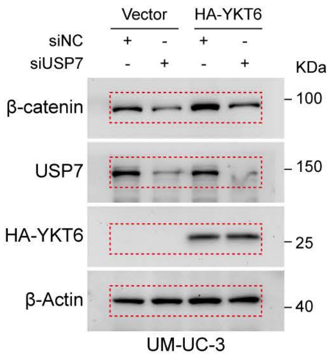

Fig. 7J

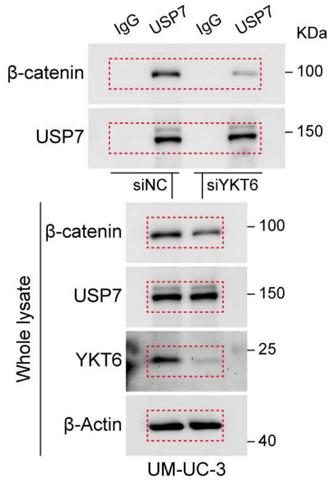

Fig. 7K

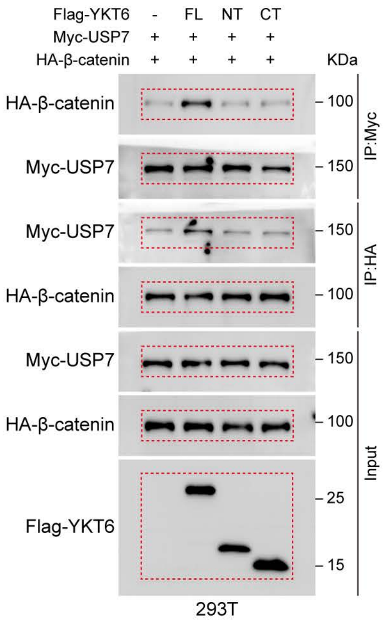

Fig. 8A

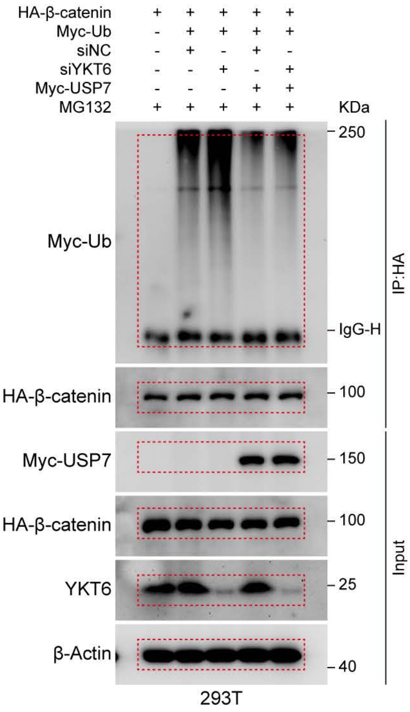

Fig. 8B

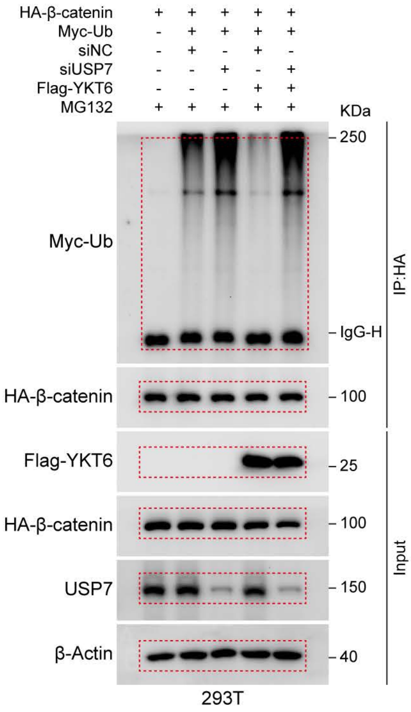

Fig. 8D

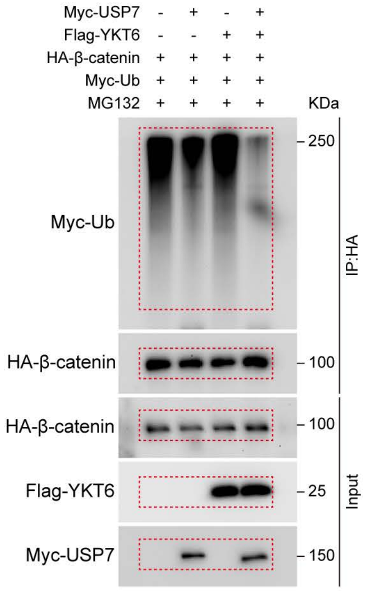

Fig. 8E

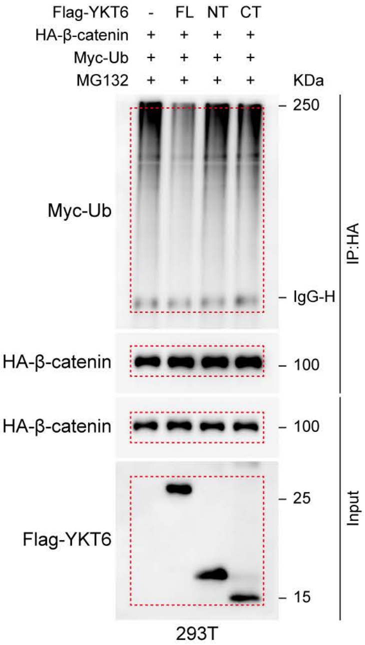

Fig. 8F

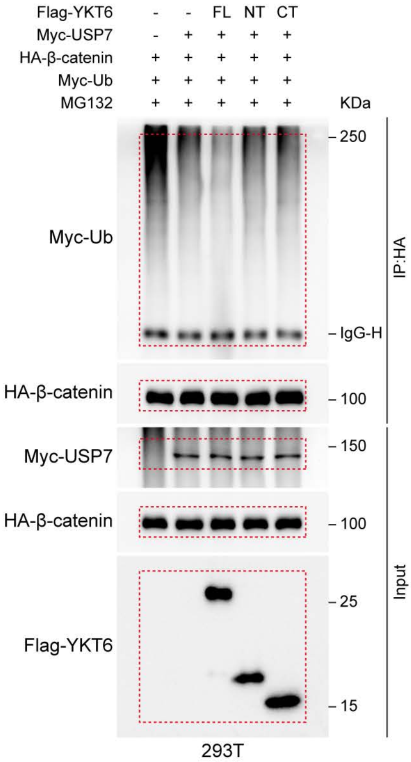

Fig. 8G

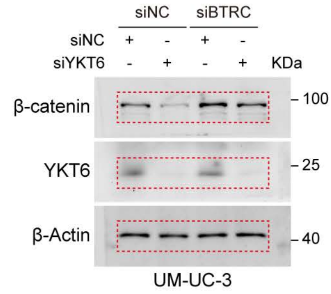

Fig. 8H

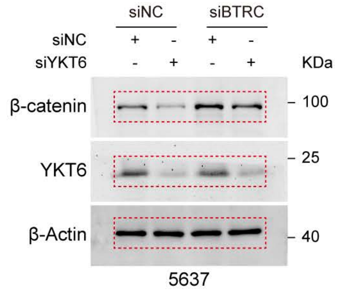

Fig. 8I

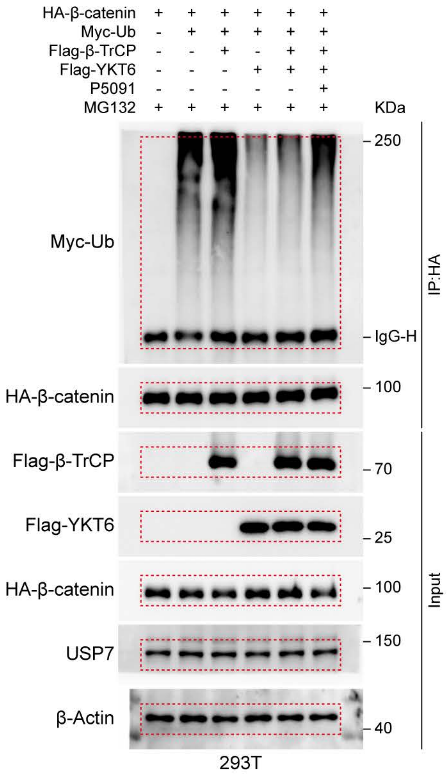

Fig. 9H

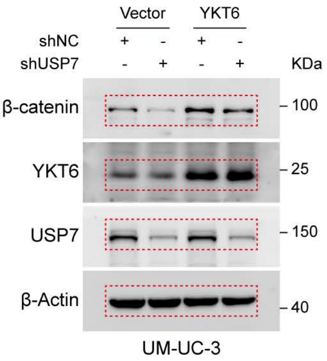

Fig. S2G

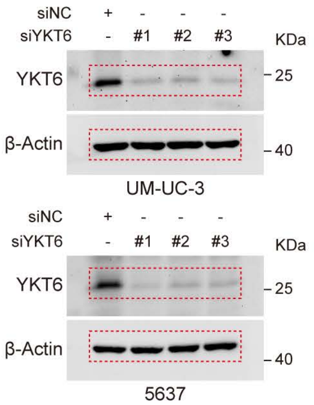

Fig. S2H

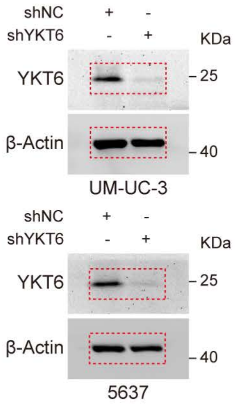

Fig. S2I

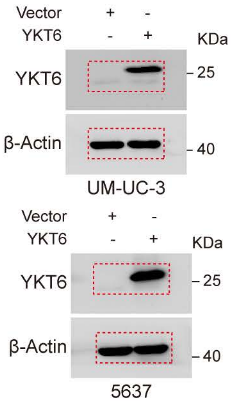

Fig. S3G

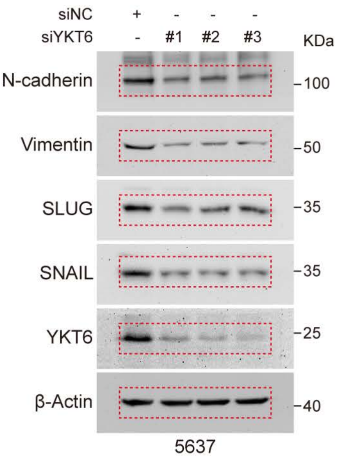

Fig. S3H

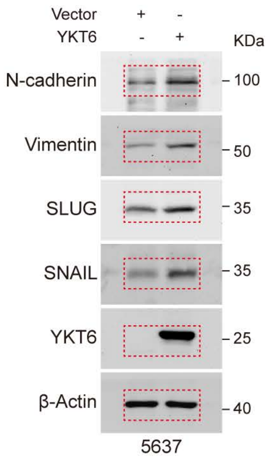

Fig. S4L

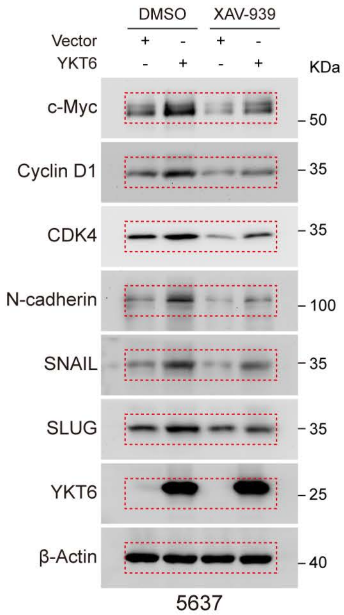

Fig. S4M

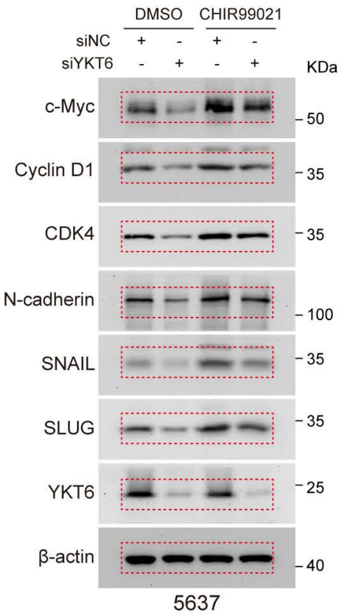

Fig. S5A

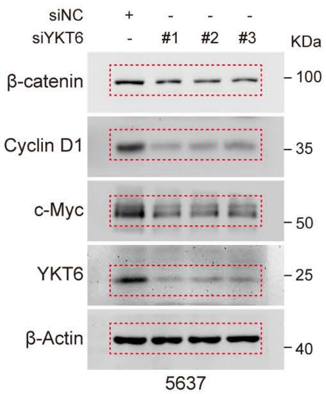

Fig. S5B

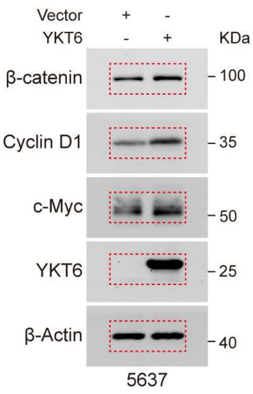

Fig. S5C

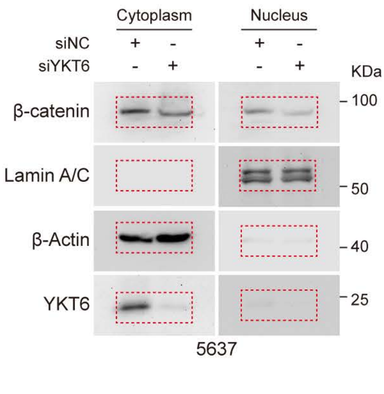

Fig. S5D

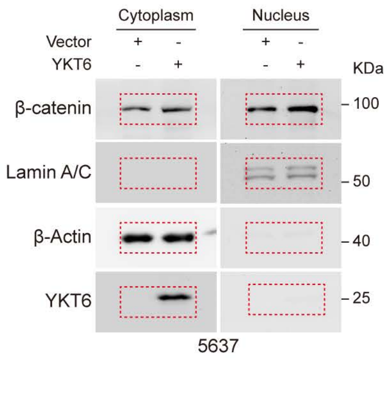

Fig. S5E

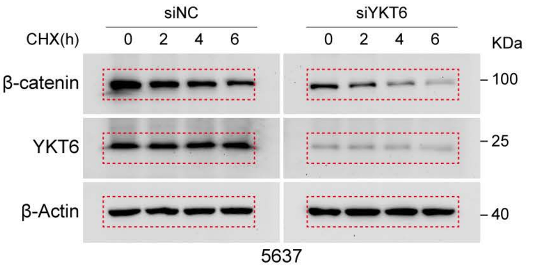

Fig. S5G

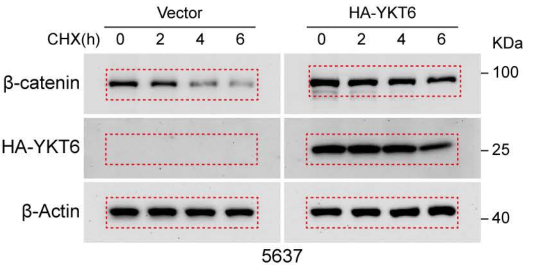

Fig. S5I

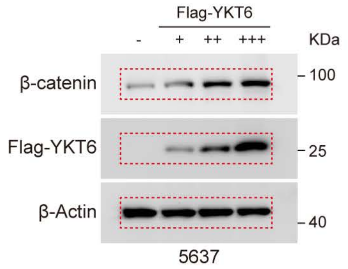

Fig. S5J

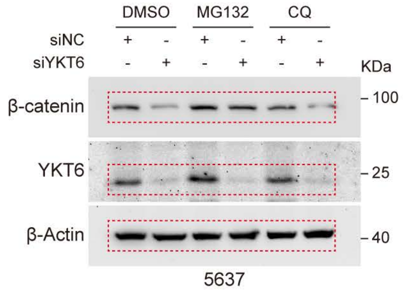

Fig. S5K

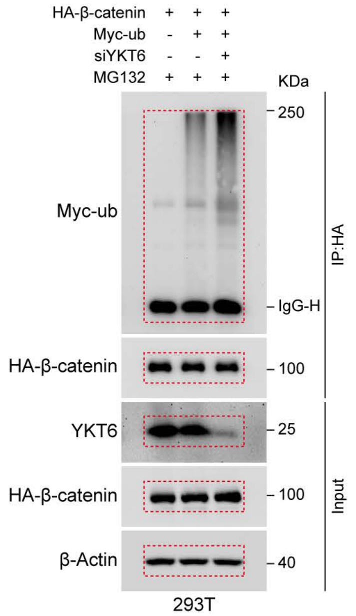

Fig. S5L

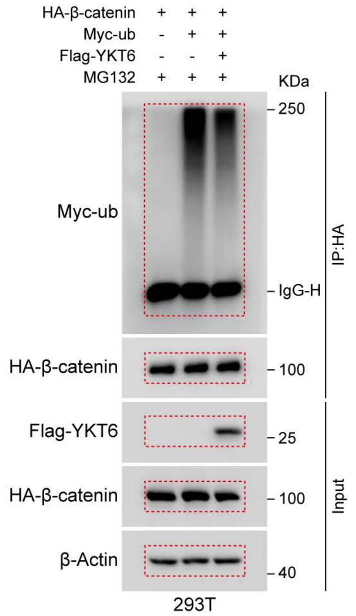

Fig. S5M

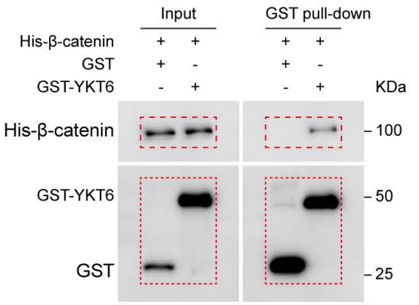

Fig. S6A

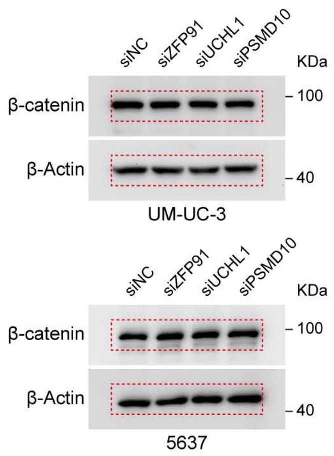

Fig. S6B

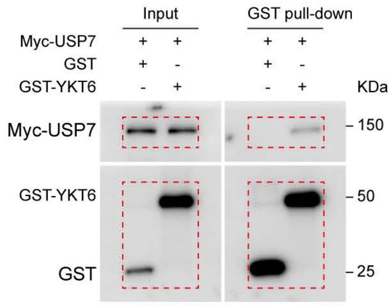

Fig. S6C

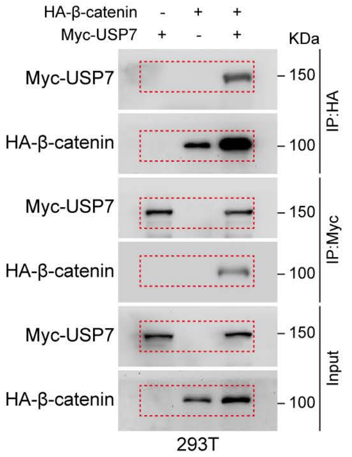

Fig. S6D

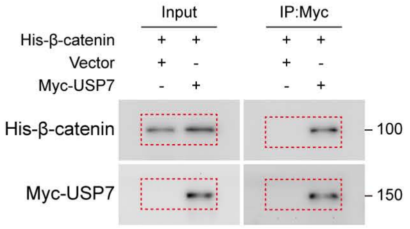

Fig. S6E

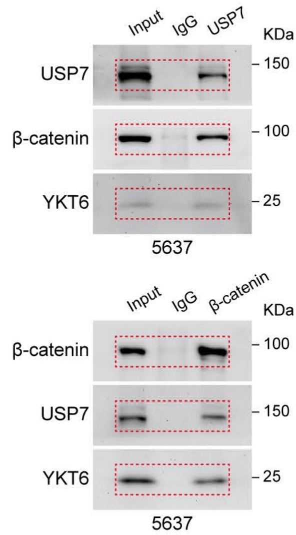

Fig. S6F

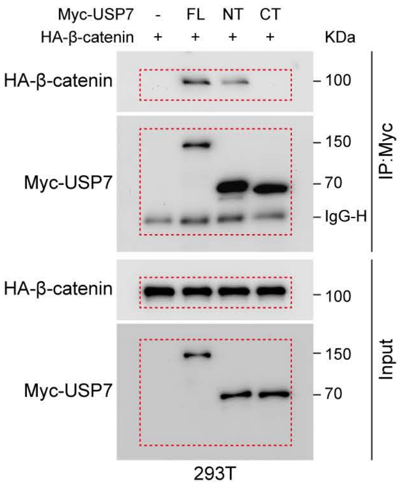

Fig. S6G

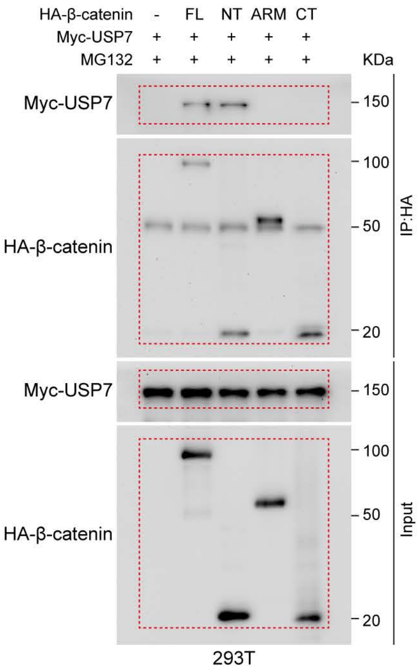

Fig. S6H

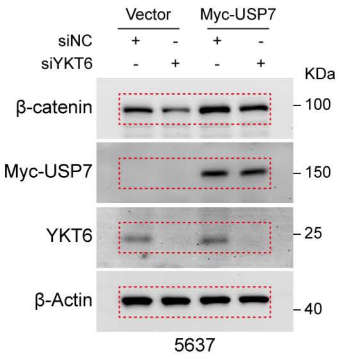

Fig. S6I

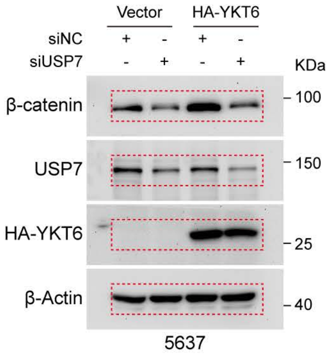

Fig. S6J

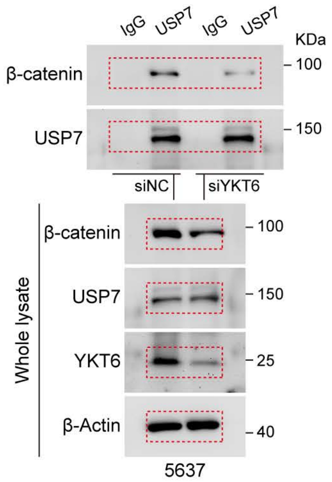

Fig. S6K

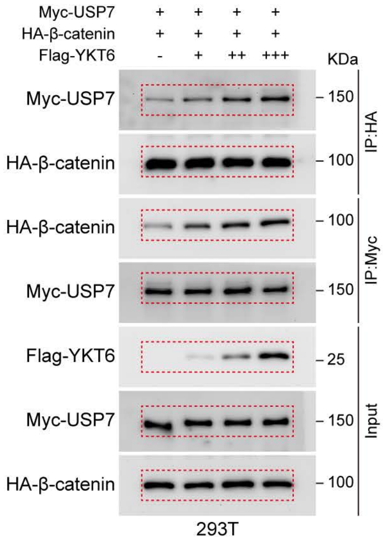

Fig. S7A

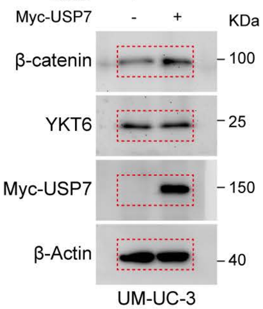

Fig. S7B

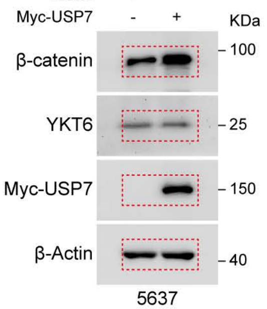

Fig. S7E

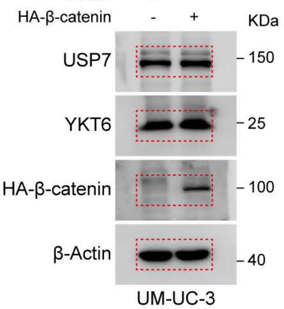

Fig. S7F

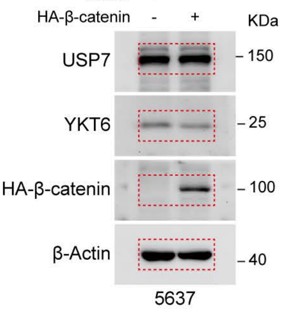

Fig. S8A

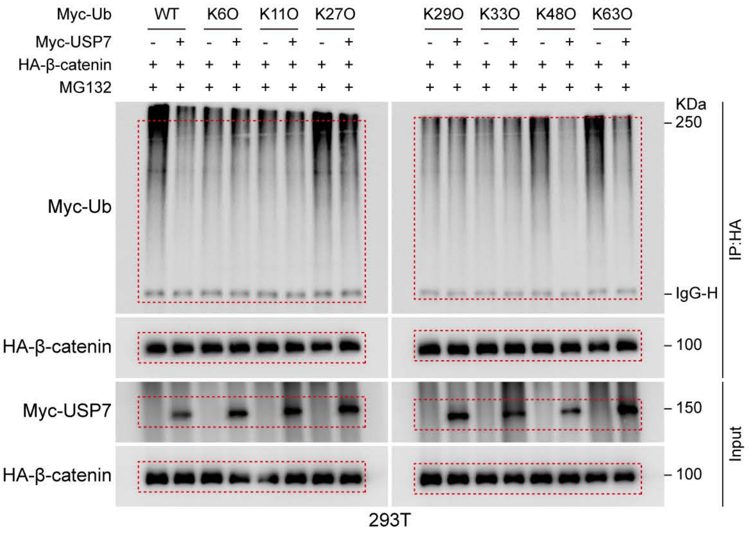

Fig. S8B

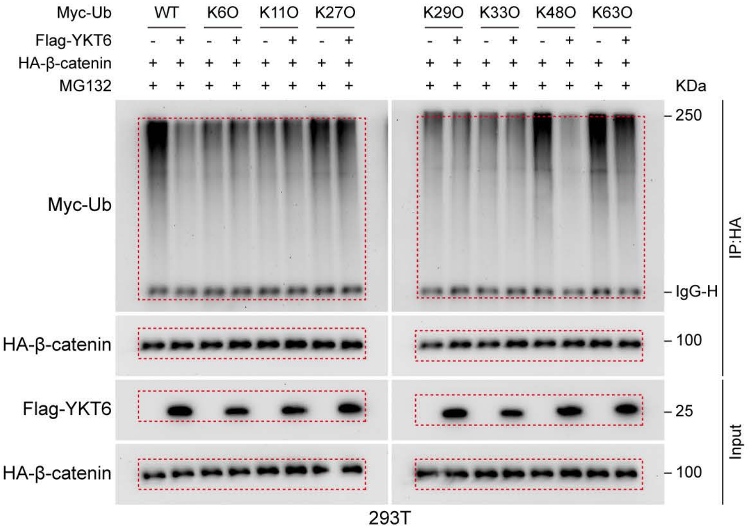

Fig. S8E

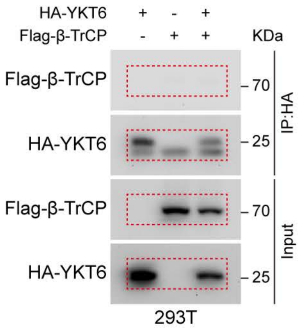

Fig. S8F

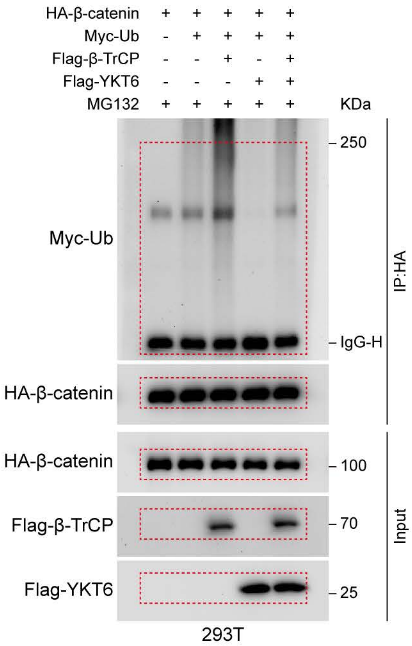

Supplement: Supplementary file 2 — Supporting Information [file ADVS-13-e07166-s001.zip › advs73011-sup-0002-data/Original Blots Images.pdf]
